# Harborview Burns – 1974 to 2009

**DOI:** 10.1371/journal.pone.0040086

**Published:** 2012-07-05

**Authors:** Loren H. Engrav, David M. Heimbach, Frederick P. Rivara, Kathleen F. Kerr, Turner Osler, Tam N. Pham, Sam R. Sharar, Peter C. Esselman, Eileen M. Bulger, Gretchen J. Carrougher, Shari Honari, Nicole S. Gibran

**Affiliations:** 1 Division of Plastic Surgery, Department of Surgery, University of Washington, Seattle, Washington, United States of America; 2 Department of Surgery, University of Washington, Seattle, Washington, United States of America; 3 Departments of Pediatrics and Epidemiology, University of Washington, Seattle, Washington, United States of America; 4 Harborview Injury Prevention and Research Center, University of Washington, Seattle, Washington, United States of America; 5 Department of Biostatistics, University of Washington, Seattle, Washington, United States of America; 6 Division of Trauma, Department of Surgery, University of Vermont, Burlington, Vermont, United States of America; 7 Department of Anesthesiology, University of Washington, Seattle, Washington, United States of America; 8 Department of Rehabilitation Medicine, University of Washington, Seattle, Washington, United States of America; Université de Technologie de Compiègne, France

## Abstract

**Background:**

Burn demographics, prevention and care have changed considerably since the 1970s. The objectives were to 1) identify new and confirm previously described changes, 2) make comparisons to the American Burn Association National Burn Repository, 3) determine when the administration of fluids in excess of the Baxter formula began and to identify potential causes, and 4) model mortality over time, during a 36-year period (1974–2009) at the Harborview Burn Center in Seattle, WA, USA.

**Methods and Findings:**

14,266 consecutive admissions were analyzed in five-year periods and many parameters compared to the National Burn Repository. Fluid resuscitation was compared in five-year periods from 1974 to 2009. Mortality was modeled with the rBaux model. Many changes are highlighted at the end of the manuscript including 1) the large increase in numbers of total and short-stay admissions, 2) the decline in numbers of large burn injuries, 3) that unadjusted case fatality declined to the mid-1980s but has changed little during the past two decades, 4) that race/ethnicity and payer status disparity exists, and 5) that the trajectory to death changed with fewer deaths occurring after seven days post-injury. Administration of fluids in excess of the Baxter formula during resuscitation of uncomplicated injuries was evident at least by the early 1990s and has continued to the present; the cause is likely multifactorial but pre-hospital fluids, prophylactic tracheal intubation and opioids may be involved.

**Conclusions:**

1) The dramatic changes include the rise in short-stay admissions; as a result, the model of burn care practiced since the 1970s is still required but is no longer sufficient. 2) Fluid administration in excess of the Baxter formula with uncomplicated injuries began at least two decades ago. 3) Unadjusted case fatality declined to ∼6% in the mid-1980s and changed little since then. The rBaux mortality model is quite accurate.

## Introduction

The Harborview Burn Center in Seattle, WA opened in 1974 under the leadership of P. William Curreri, M.D., Janet Marvin, R.N., M.N., Verna Cain, R.N., and Leslie Einfeldt, R.N. Collection of the demographics, injury characteristics, and outcome from acute burn injuries began at that time. We have continued this database without interruption for 36 years (1974–2009) and it now provides a unique opportunity to review changes in burn care over a long period of time, at a single center, including both children and adults, with a stable surgical and rehabilitation staff.

There have been several prior reviews of burn injury demographics, prevention, and care since the mid-1990s [1], [2], [3], [4], [5], [6], [7], [8], [9], [10], [11]. However, most were either focused on a select patient cohort or outcome, were discontinuous in time, or covered a relatively short time span. We herein report our results and include observations on changes in numbers of standard and short-stay admissions, age distribution, transport patterns, TBSA%, race/ethnicity, payer status, “fluid creep” [12], burn surgery, length of stay, mortality, trajectory to death, and “unprecedented” survival. The changes are highlighted at the end of the manuscript and the implications for burn care organization and delivery presented in the Discussion.

## Methods

### Definitions

We defined adults as ≥16 years of age and children as <16 years of age. Incidence rate was defined as the number of events per 100,000 of the relevant population per year. All incidence rates were calculated using solely admissions and populations from WA (populations are shown in File S1) where WA State refers to Washington State. In this article we classified admissions into two types. Standard Admission (Type 1): Classic burn admission of any number of days for inpatient care of complex wounds until healed or grafted. Short-Stay Admission (Type 2): Admission for one or two days for wound care, education and pain control followed by outpatient management. The revised Baux score (rbaux) is calculated as age + TBSA% +17*(presence of inhalation injury) [13] where TBSA% is total body surface area percent).

### Human Subjects

The Human Subjects Division of the University of Washington approved this study (#34999).

### Data Sources

All patients (n = 14,266) admitted to the Harborview Burn Center with new cutaneous burn injuries from all etiologies between 8/2/1974 and 12/31/2009 were prospectively entered into a database. Those immediately placed on comfort care on arrival were included whereas persons with only smoke inhalation (no cutaneous injury) were excluded. The data was collected by various faculty/staff over the decades and the data points were those reported below. Prior to analysis, the entire database was searched for missing and out of range values and the data obtained or corrected from the medical records, if possible. The final result is that the maximum number of missing values was 305/14266 (2.1%) for payer status. The percent of missing values for all other parameters reported was <2.1%.

Several other datasets were also used in this analysis. The Comprehensive Hospital Abstract Reporting System (CHARS) administered by the WA State Department of Health [14] was used to obtain the number of admissions in WA State. Populations for WA State were obtained from HistoryLink.org [15]. Populations of the several race/ethnicities were obtained from the Office of Financial Management of WA State [16] and HistoryLink.org [15]. WA State Medicaid enrollment was obtained from the WA State Department of Social & Health Services Research & Data Analysis Division [17]. Numbers of admissions of burn injuries in the United States from 1994 to 2009 were obtained from the Healthcare Cost & Utilization Project (HCUP) [18].

Comparisons of our historical database were made to the ABA-NBR (American Burn Association National Burn Repository, American Burn Association, Chicago) using data obtained in April 2011 (version 6.0). The ABA-NBR includes data all persons admitted to 94 voluntarily participating U.S. burn centers. However, since not all hospitals participate, it is a convenience sample and does not include all persons or a random sample of all persons admitted to hospitals or burn centers for care of burn injuries [19].

The ABA-NBR dataset contained 280,664 records after excluding those from the Harborview Burn Center. Harborview records were excluded from our analysis to ensure comparison to other centers. As the ABA-NBR records included admissions for isolated inhalation injury, other skin diseases, and readmissions for complications or reconstruction, we used various filters (see File S2) to identify patients with new cutaneous burns, as recommended by Pavlovich [20].

We also created several subsets of the total ABA-NBR where all variables of interest were recorded, to maximize the number of records used for each comparison. For example, if a record contains race/ethnicity data but not smoke inhalation data, it can be used in race/ethnicity comparisons and ought not be removed from all analyses.

The Overall Set of the ABA-NBR included records for new burn admissions with known etiology, age, TBSA%, survival status and year of injury (File S3). There were too few records prior to 1995 to permit comparison of earlier periods, 1995–2009 was simply divided into five-year time periods, 1995–1999 (n = 27,891), 2000–2004 (n = 38,104) and 2005–2009 (n = 55,257) (File S3). Comparisons to Harborview data were only done after 1994.

The Large Burn Set is a subset of the Overall Set restricted to records with TBSA >20% from thirteen facilities that submitted consistent data from 1995 to 2009; it contained 4,538 records from 1995–1999 (n = 1,771), 2000–2004 (n = 1,638) and 2005–2009 (n = 1,129) (File S3).

The Mortality Regression Set is another subset of the Overall Set restricted to those records with known survival status, presence of inhalation injury, sex and race/ethnicity and includes 102,669 records from 1995–1999 (n = 25,773), 2000–2004 (n = 33,000) and 2005–2009 (n = 43,896) (File S3). In this analysis we divided persons into three groups: known to have died, discharged alive, and unknown survival status. The true mortality is likely somewhat higher as some deaths attributed to a person’s burn injury may have occurred after discharge/transfer from the burn center.

The Mortality Model Set is another subset of the Overall Set restricted to those records with known inhalation status and year >1994 and contained 113,149 records from 1995–1999 (n = 27,760), 2000–2004 (n = 36,379) and 2005–2009 (n = 49,010) (File S3).

The Length of Stay set is the final subset of the Overall Set restricted to those records with known length of stay and contained 120,046 records from 1995–1999 (n = 27,805), 2000–2004 (n = 37,989), and 2005–2009 (n = 54,252) (File S3).

### Fluid Resuscitation

For the fluid resuscitation analysis, a subset of the Harborview database was examined. Fluid requirements are substantially influenced by several variables including burn size, age, type of injury (e.g. electrical), presence of inhalation injury, etc. An extremely large sample size would be required to study first 24-hour fluid resuscitation and associations with all of the important variables. Furthermore it is unclear if “fluid creep” [12] is present in resuscitation of uncomplicated injuries. Accordingly, we limited the fluid analysis to persons who survived to discharge with TBSA% from 20–60%; age ≥14 years; flame, scald or flash etiology; no inhalation injury and no other injuries. We asked three questions 1) was “fluid creep” present in successful resuscitation of uncomplicated injuries, and if so, 2) approximately what year did the practice begin, and finally, 3) what therapeutic events are associated with “fluid creep”?” There were 768 records that met these criteria from 1974 to 2006 and the list was submitted to Harborview Department of Health Information Management for retrieval. The timespan from 1974 to 2009 was divided into 5-year time periods and record retrieval continued until each 5-year time period contained at least fifteen complete records. Opioid equivalents were calculated as morphine 10 mg, hydromorphone 1.5 mg, fentanyl 100 µg, and methadone 5 mg.

### Mortality Modeling

Many models to predict mortality for burn injury have been published over the years. Most include parameters not present in our historical database, such as pneumonia [21], percent full-thickness injury [22] and Abbreviated Burn Severity Index [23], and day one physiologic data FLAMES score [24]). In 2010 Colohan [25] reviewed previously described models of mortality and concluded that TBSA%, presence of inhalation injury, and age are the strongest predictors of mortality. The Belgian Outcome in Burn Injury Study Group studied 5,246 burn injured persons and published an elegant, simple method to predict burn mortality [26] based upon the method of Ryan [27] and the modified model was validated by Brusselaers [28]. However the definition of inhalation injury in the model is that mechanical ventilation was required. Our database includes a much broader definition of inhalation injury so the Belgian model could not be applied. The Osler [13] rBaux model fulfills the requirements of Colohan [25] and is applicable to our database.

Osler [13] published the rBaux Score using age, TBSA% and inhalation based upon 39,888 records from 2000–2007 in Version 4.0 of the ABA-NBR dataset. The rBaux score is calculated as age + TBSA% +17*(presence of inhalation injury). This means, for example, that an rBaux score of 117 might refer to a 50 year old person with 50% TBSA% and inhalation injury, a 25 year old person with 75% TBSA% and inhalation injury, a 50 year old person with 67% TBSA% and no inhalation injury, etc.

The predicted mortality may be calculated with the equation below. We segregated the rBaux scores into seven groups for this analysis, (1) ≤75, (2) >75 and ≤85, (3) >85 and ≤100, (4) >100 and ≤115, (5) >115 and ≤130, (6) >130 and ≤150 and (7) >150. The groups were chosen based upon the distribution of observed mortality versus rBaux score presented by Osler [13]. The predicted mortality range for each group is calculated with Equation 1.
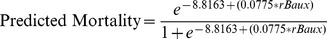
(1)


### Statistical Analysis

As discussed above, the ABA-NBR is not a representative sample of the United States burn population. Neither is the Harborview data a representative sample of the larger population. Therefore, we considered both to be a population, for which we have complete data, not a sample of the United States population of burn patients. Therefore analyses, including changes over time, are done with descriptive, not inferential, statistics. Linear regression was used to assess trends in fluid administration, quantile regression to assess trends in length of stay, and logistic regression to assess risk factors for mortality. To further assess trends in mortality, we used a moving average of the observed/expected mortality ratio using a moving window of 2,000 patients, which corresponded to about two years of calendar time. Data analysis was accomplished with STATA version 11.1 (StataCorp, TX).

## Results

Some of the results invite comment/explanation that does not warrant inclusion in the Discussion section; these are included here in Results.

### Catchment Area

**Figure 1:**
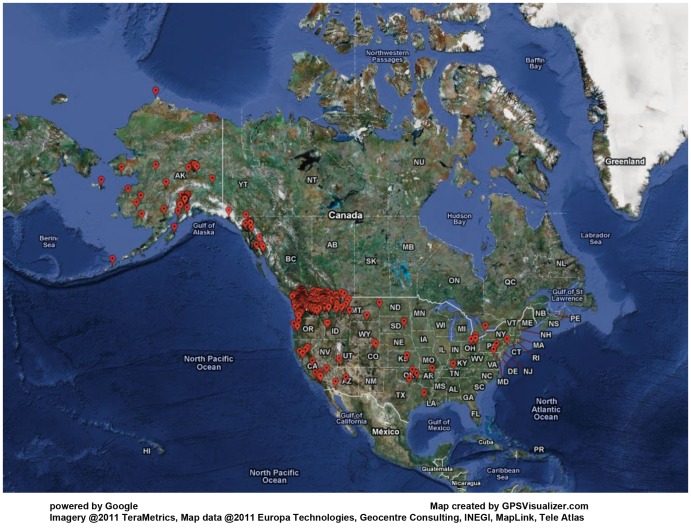
Catchment Area 2005–2009. The majority of referrals come from Alaska, WA, the panhandle of Idaho and western Montana.

The majority of Harborview admissions were referred from WA State; other admissions came from Alaska, the panhandle of Idaho, and western Montana. Some persons from elsewhere sustained their injury in the Seattle region and were treated in the burn center. The catchment area did not change from 1974 to 2009. The zip code of residence of the 3,252 persons admitted in 2005–2009 is shown in Fig. 1.

### Admissions

Upon analysis of the database, it became clear that there are two types of admissions to the Burn Center. Standard (Type 1) is the classic admission requiring complex inpatient care of any number of days. Short-stay (Type 2) is a short admission (defined as one or two days with survival) for initial wound care, pain control and education; the patient is then discharged to outpatient care.

For this analysis we defined Short-stay as one or two days determined after discharge, upon review of the data. This clearly means that some persons classified as Standard (Type 1) could have been Short-stay (Type 2) had they lived nearby the Harborview Burn Center or had sufficient family support. It also means that persons with “simple” injuries that require surgery might now be Short-stay (Type 2) whereas in the past they might have been Standard or Type 1. But neither of these variables is new and should not affect comparison of changes over time.

The concept of the two types of admissions is not new. Although the authors did not use these terms, Davey mentioned such a distribution over a decade ago [4] and more recently Anwar [29] and Ward [30]. The two types of admissions are very different, e.g. our overall mortality was 7.2% for standard admissions and zero for short-stay, median TBSA% was 8.0% for standard admissions and 2.5% for short-stay, and median rBaux score [13] was 42 for standard admissions and 25 for short-stay.

Nevertheless, studies do not typically differentiate between standard and short-stay admissions; an exception is the report by Onarheim [31]. This was unimportant in the 1970s and 1980s as the numbers of short-stay admissions were small. They did not become significant until the 1990s and have been increasing since then, now comprising greater than 50% of admissions to Harborview and ∼40% of records in the Overall Set of the ABA-NBR. Combining the two types of admissions renders historical comparison to the 1970s and 1980s impossible and interpretation difficult. It seemed prudent to segregate the two types of admissions and exclude short-stay admissions from reports on mortality, length of stay, etc. This addresses the issue raised by Jeng [32], i.e. the need to filter the noise to see the signal. We will discuss the two types of admissions separately in the analyses that follow.

#### Standard admissions (Type 1)

**Figure 2:**
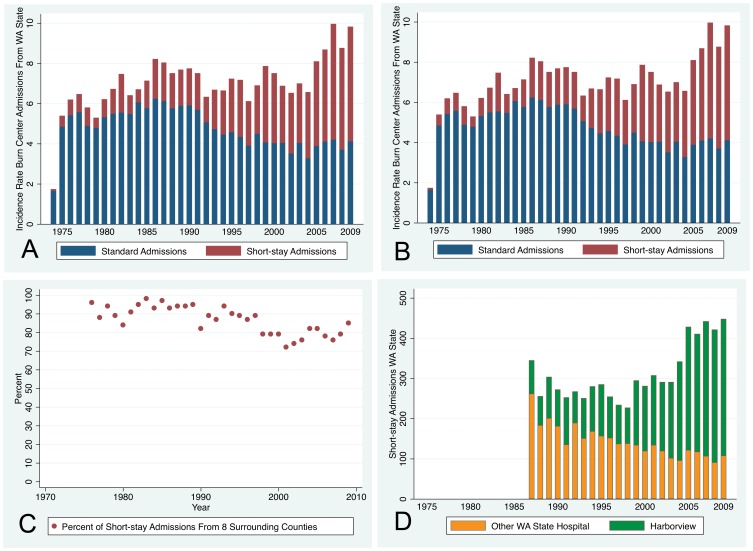
Admissions Over Time. Panel A – The number of standard (Type 1) admissions was stable over the several decades. (1974 was a partial year) Panel B – The incidence rate of standard admissions declined from the mid-1980s to the mid-1990s and has been stable since then. (1974 was a partial year) Panel C – The majority (72–98%) of the short-stay admissions originated in the eight WA State counties surrounding Seattle with a slight decline over time. Panel D – There was a decline in short-stay admissions (Type 2) to other WA State hospitals and an increase to Harborview.

The number of standard admissions to Harborview was quite stable over the several decades (Fig. 2, Panel A). However, the incidence rate of standard admissions peaked in the mid-1980s, declined to the approximately year 2000, and has been stable since then (Fig. 2, Panel B).

Standard admission records in the Overall Set of the ABA-NBR rose from 20,649 in 1995–1999 to 24,030 in 2000–2004 and to 33,677 in 2005–2009. Since the ABA-NBR is based on voluntary submission, these figures do not necessarily indicate an increasing incidence rate of burn injuries; more likely they indicate increasing submission.

#### Short-stay admissions (Type 2)

On the contrary, the number of short-stay admissions to Harborview increased over time (Fig. 2, Panel A and D), as did the incidence rate (Fig. 2, Panel B). In 2005–2009 short-stay admissions made up 53% of admissions. The majority of short-stay admissions originated in eight counties surrounding Seattle (Fig. 2, Panel C) and could therefore be followed as outpatients, unlike those who traveled a great distance. Over the same period short-stay admissions to other WA State hospitals declined (Fig. 2, Panel D).

The proportion and absolute number of short-stay admissions has also risen in the Overall Set of the ABA-NBR. There were 7,242 in 1995–1999, 14,074 in 2000–2004 and 21,580 in 2005–2009. This constitutes 26%, 37% and 39% of records respectively in the Overall Set of the ABA-NBR.

#### Comment on admissions

It seems likely that the decline in incidence of standard admissions to Harborview up to approximately year 2000 was part of the national trend. HCUP data [18] reports ∼50,000 hospital discharges for burn injury in 1994 declining to ∼30,000 in1999 and remaining stable since then. That the incidence rate of standard admissions has been steady since the mid-1990s suggests no further improvement in the prevention of large injuries during the past 15 years. A similar pattern was found in Norway [31] in a study in which the investigators did attempt to limit the study to standard admissions. Åkerlund [33] reported a similar pattern in Sweden and Spinks [34] in Canada. These observations suggest that further reduction in serious burn injuries will require new prevention strategies.

In contrast to standard admissions, short-stay admissions have increased substantially at Harborview and in the ABA-NBR. One can only speculate on the reasons for the increasing numbers and incidence rate of short-stay admissions; they are probably many and complex. Anwar [29] and Ward [30] described similar changes in the UK. It is possible that the number of such injuries is increasing. More likely, it may be that less serious injuries are being referred to specialty centers in greater proportions, following the “nationwide movement toward regionalized multidisciplinary care” [35], [36]. This hypothesis is supported by the decline in burn admissions to other WA State hospitals. Burn care has become quite complex and is an elective in many training programs. Therefore burn care may exceed the capability of many physicians and surgeons. In addition since Medicaid covers ∼40% of those ≤15 years and 15–35% of those ≥16 years, it is possible that burn injuries are referred for financial considerations. Finally, the Harborview transfer center opened in the late 1990s and has increasingly facilitated referrals; this may have also played a role in the increased numbers of admissions.

### Percent Total Body Surface Area (TBSA%)

#### Standard admissions (Type 1)

**Figure 3:**
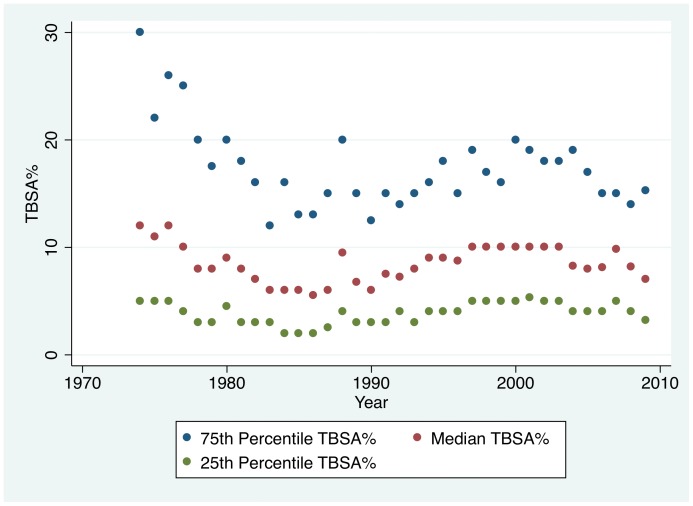
TBSA% Over Time for Standard Admissions. The median TBSA% declined to the mid-1980s and subsequently varied from 7% to 10%. The 75^th^ percentile TBSA% also declined to the mid-1980s and then varied from 12% to 20%. The 25^th^ percentile was stable between 3% and 5%.

For standard admissions, the median TBSA%, 25^th^ percentile, and 75^th^ percentile declined to the mid- to late-1980s. Since the early 1990s the median TBSA has been ∼10%, 25^th^ percentile ∼5%, and 75^th^ percentile ∼15% (Fig. 3). In 2005–2009, the most recent period, the median TBSA was 8% (IQR 4–15), 36% of admissions were ≤5% TBSA, and 3% of admissions were ≥60% TBSA. ABA-NBR data for standard admissions were very similar.

#### Standard admissions (Type 1) with TBSA% >20%

**Figure 4:**
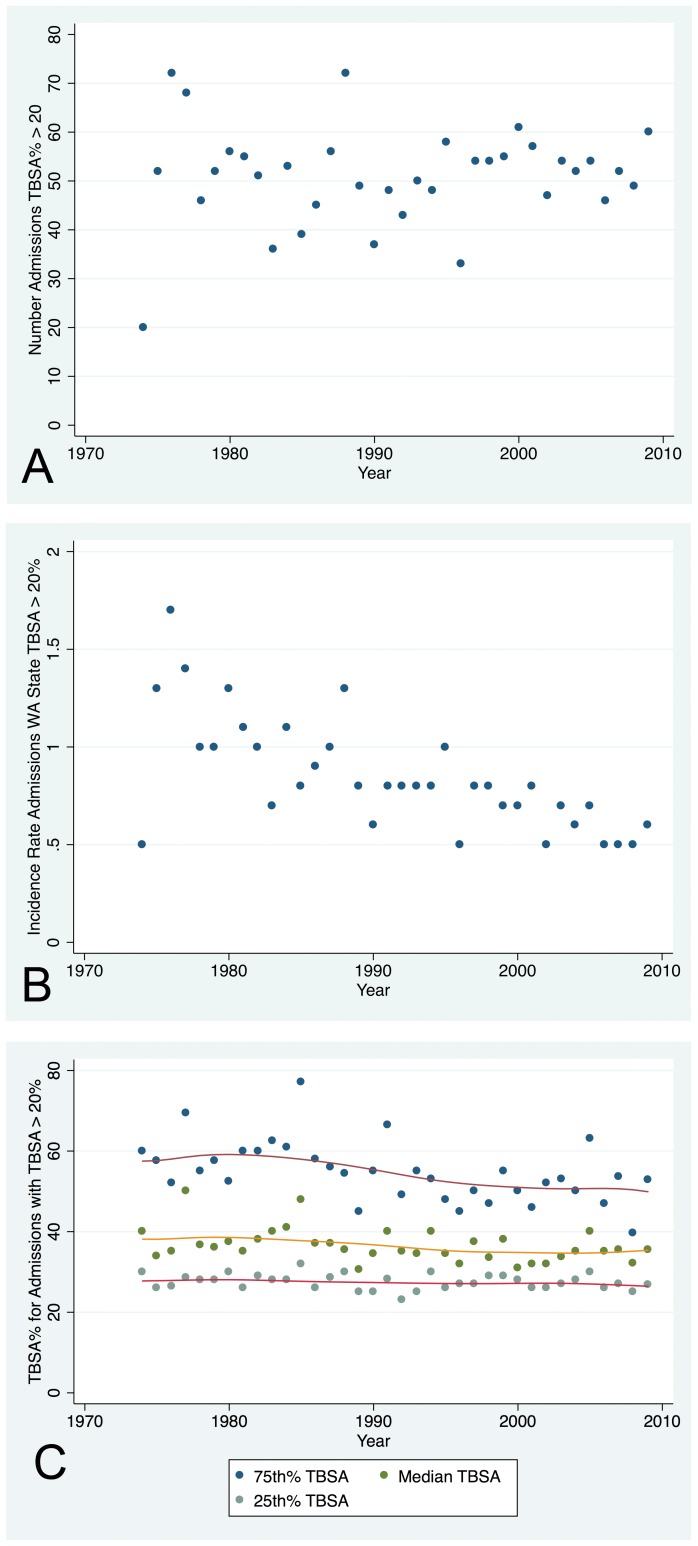
Admissions with TBSA% >20%. Panel A – There was no trend over time to the number of admissions per year with TBSA% >20%. Panel B – The incidence rate of admissions with TBSA >20% declined from approximately 1.5 in the late 1970s to approximately 0.5 in 2009. Panel C – The 75^th^ percentile and median TBSA% declined modestly over time: 75^th^ percentile: ∼60 to 50% and median: ∼40 to 35%.

There was no trend over time to the number of admissions per year with TBSA greater than 20%; the number varied around 50 (Fig. 4, Panel A). However, the incidence rate of admissions with TBSA >20% declined over time (Fig. 4 Panel B). The 75^th^ percentile TBSA% and the median declined modestly; there was little change in the 25^th^ percentile (Fig. 4 Panel C).

There was no trend to the number of admissions with TBSA greater than 20% at Harborview, however this was not the case in the ABA-NBR. From the Large Burn Set of the ABA-NBR based upon thirteen centers, the number of records with TBSA% >20% declined from average 354 per center in 1995–1999 (71 per year), to 328 in 2000–2004, and to 225 per center in 2005–2009 (45 per year).

#### Short-stay admissions (Type 2)

The median TBSA% of short-stay admissions did not change over time and was 2% (IQR 1–5%).

#### Comment on TBSA%

TBSA% of standard admissions declined until the mid- to late-1980s but has been stable since the early 1990s. The number of admissions with large injuries has also been stable even though the incidence rate is declining.

### Age

#### Standard admissions

For standard admissions to Harborview the median age increased from a 1975–1979 median of 28 (IQR 15–47) to a 2005–2009 median of 37 (IQR 20–52). The number of admissions in age groups 46–65 and >65 years increased more than age groups ≤5, 6–15 and 16–45 explaining the increase in median age.

Children accounted for ∼30% of standard admissions until 1995–1999 but this percentage declined to ∼20% in 2000–2004 and 2005–2009. Their median age was 2 (IQR 1–8) and 67% were ≤5 years, confirming that younger children are at greater risk of injuries requiring standard admission than older children. Persons over 65 years made up ∼8% of standard admissions in the early 1980s and ∼10% in 2005–2009.

The incidence rate of standard admissions for children rose from ∼5/100,000/year in the late 1970s to ∼8 in 1990 and then declined to ∼4 in the late 1990s and remained there. The incidence rate of standard admissions for persons >65 years was stable at 4–5 over the 36 years.

Patterns in the Overall Set of the ABA-NBR were quite similar including the decline in the proportion of standard admissions of children.

#### Short-stay admissions

The median age of short-stay admissions was stable over the entire 36-year period and in 2005–2009 was 21 (IQR 3–40), younger than standard admissions. Children accounted for ∼40% of admissions since the mid-1980s, twice the percentage of standard admissions. The median age of children was 2 years, the same as for standard admissions, and 74% of the children were ≤5 years. Younger children were also at greater risk for injuries resulting in short-stay admissions than older children. Persons over 65 years made up ∼2% of these admissions for the same period, fewer than for standard admissions.

Patterns in the Overall Set of the ABA-NBR were quite similar.

#### Comment on age

The increase in the median age of persons with standard admissions is likely a reflection of the aging of the population as a whole; the median age in WA State was ∼27 in 1975 and ∼40 in 2010. Similarly the proportion of children among standard admissions fell from ∼30% in 1975 to ∼20% in 2009, while their proportion in the general population fell from ∼28% in 1975 to ∼19% in 2010. The proportion of standard admissions for persons ≥65 years was quite stable at ∼10% and their numbers in the general population only changed from ∼10% in 1975 to ∼12% in 2010.

The incidence rate for children declined from 1990 to 2000 but has changed little since. Bowman [37] found a similar pattern nationwide in the United States. The incidence rate of admission for persons >65 years was stable over the 36 years.

### Sex

The sex distribution did not change over time for either standard or short-stay admissions. However, there was difference in the sex distribution by age group. Of those persons ≤5 and >65 years, ∼60% were male; ∼75% were male between 6 and 65 years. The same pattern exists in the Overall Set of the ABA-NBR.

### Race/Ethnicity

Rivara [38], co-author of this manuscript, has pointed out that race and ethnicity should not be used as explanatory variables when the underlying constructs (e.g. education, income) can be measured directly. However, he also states that, when considering health disparities including demographics and outcomes, it is appropriate to use race and ethnicity, not as explanatory variables, but to examine the underlying sociocultural reasons for the disparities. It is in this sense that we include self-declared race and ethnicity.

The categorization of race/ethnicity changed in 2000 when the United States Census Bureau subdivided each race into Hispanic and non-Hispanic ethnicities and more recently when the Department of Health and Human Services adopted new standards for recording race/ethnicity [39]. The Harborview database and the ABA-NBR do not include this detail, rather they use the categories previously defined by the Census Bureau including White, Black, Pacific Islander, Asian, Native American, Hispanic and Multiracial. This introduces some discrepancy but the changes were not sufficiently large to alter conclusions.

For standard admissions to Harborview, the proportion of Whites and Blacks decreased over time whereas that of Hispanic, Asian, and non-White increased for both children and adults (Table 1). For standard admissions of children the incidence rate for Whites, Blacks and non-Whites declined whereas the rate rose for Hispanics and Asians (Table 1). The incidence rate was higher for Black, Hispanic, Asian, and non-White children than White. For standard admissions of adults, the incidence rate rose for Hispanics but declined for the other groups (Table 1). The incidence rate was higher for Blacks than the other groups.

**Table 1:** Race/Ethnicity of Standard Admissions.

Proportion					
Children	White	Black	Hispanic	Asian	Non-White
1975–1979	74%	17%	2%	1%	21%
1980–1984	69%	15%	5%	7%	27%
1985–1989	70%	14%	5%	6%	25%
1990–1994	75%	10%	5%	6%	21%
1995–1999	66%	8%	15%	6%	29%
2000–2004	64%	7%	16%	5%	29%
2005–2009	53%	11%	21%	8%	40%
**Adults**	**White**	**Black**	**Hispanic**	**Asian**	**Non-White**
1975–1979	83%	8%	1%	2%	11%
1980–1984	80%	8%	3%	3%	15%
1985–1989	81%	8%	3%	5%	16%
1990–1994	81%	7%	5%	4%	16%
1995–1999	81%	5%	5%	4%	14%
2000–2004	84%	4%	5%	3%	13%
2005–2009	77%	5%	8%	5%	18%
**Incidence Rate**					
**Children**	**White**	**Black**	**Hispanic**	**Asian**	**Non-White**
1975–1979	4.3	41.4	5.0	2.9	15.6
1980–1984	4.9	36.5	9.3	12.7	18.2
1985–1989	5.7	36.0	9.8	11.2	17.5
1990–1994	6.2	23.4	7.2	10.6	12.4
1995–1999	4.5	14.3	14.7	6.7	11.8
2000–2004	3.4	8.9	8.5	3.9	7.0
2005–2009	3.2	14.5	10.5	5.5	9.4
**Adults**	**White**	**Black**	**Hispanic**	**Asian**	**Non-White**
1975–1979	5.1	19.4	2.6	4.8	8.6
1980–1984	5.1	18.3	5.3	4.9	8.8
1985–1989	5.5	18.1	4.6	7.2	9.2
1990–1994	4.8	12.0	5.1	4.1	6.5
1995–1999	4.3	7.2	4.0	3.6	4.6
2000–2004	4.8	5.9	2.9	2.5	3.3
2005–2009	4.8	7.2	4.4	3.4	4.5

The proportion of Whites and Blacks decreased over time whereas that of Hispanic, Asian, and non-White increased for both children and adults. For children the incidence rate for Whites, Blacks and non-Whites declined whereas the rate rose for Hispanics and Asians. The incidence rate was higher for Black, Hispanic, Asian, and non-White children than White. For adults, the incidence rate rose for Hispanics but declined for the other groups. The incidence rate was higher for Blacks than the other groups. (The row totals do not equal 100% since some categories with small n were omitted from the table).

The trends were similar for Harborview short-stay admissions. On the contrary, in the Overall Set of the ABA-NBR from 1995 to the present for both standard and short-stay admissions, the proportions remained stable. For standard admissions of children in the ABA-NBR the proportions are White ∼40%, Black ∼20%, Hispanic ∼20%, and Asian ∼5%. For standard admissions of adults the proportions are White ∼60%, Black ∼20%, Hispanic ∼10%, and Asian ∼5%.

#### Comment on race/ethnicity

The race/ethnicity distribution is strikingly different from other centers/regions. Garner [8] reported 56% Hispanic and McGwin [40] ∼30% Blacks. This regional difference could have implications for multi-center trials.

The incidence rate was higher for persons of color. Karr previously reported this discrepancy for Hispanic children [41] as did Rimmer [42]. There continues to be a disparity in the proportions and incidence rates for persons of color.

### Payer Status on Admission (relevant only to USA readers)

#### Children

The proportions of payer status on admission for standard and short-stay admissions were similar and shown overall in Table 2. Medicaid coverage rose to nearly 50% of admissions in the early 1990s, then decreased, and has now risen again to similar levels. The current distribution (2005–2009) is ∼45% Medicaid, ∼45% Insurance and ∼5% uninsured.

**Table 2:** Payer on Admission by Age.

Children	Medicare	Medicaid	Insurance	L&I	Uninsured
1975–1979		37%	50%		9%
1980–1984		38%	49%		11%
1985–1989		39%	41%		10%
1990–1994		49%	41%		2%
1995–1999		27%	52%		5%
2000–2004		27%	60%		5%
2005–2009		44%	44%		6%
**Adults**	**Medicare**	**Medicaid**	**Insurance**	**L&I**	**Uninsured**
1975–1979	14%	17%	32%	23%	11%
1980–1984	11%	23%	31%	20%	13%
1985–1989	11%	27%	25%	24%	10%
1990–1994	12%	40%	22%	20%	4%
1995–1999	14%	29%	24%	18%	9%
2000–2004	14%	15%	27%	16%	22%
2005–2009	14%	16%	29%	18%	19%

For those ≤15 years, Medicaid coverage rose to nearly 50% in the early 1990s, then decreased, and has now risen again to similar levels. Insurance coverage followed a reversed pattern. The current distribution (2005–2009) is ∼45% Medicaid, ∼45% Insurance and ∼5% Uninsured. For those ≥16 years, Medicaid coverage increased to 40% in the early 1990s and then declined. Insurance coverage declined to the early 1990s and has now risen. The proportion of L&I coverage has declined over time but has changed little since the early 1990s. Medicare coverage has been quite stable. The current distribution (2005–2009) is ∼15% Medicare, ∼15% Medicaid, ∼30% Insurance, ∼20% L&I and ∼20% Uninsured. (The row totals do not equal 100% since some categories with small n were omitted from the table.)

The ABA-NBR proportions of payer status for standard and short-stay admissions of children followed a similar, but less striking, pattern. Medicaid coverage declined from 25% in 1995–1999 to 22% in 2000–2004, and then rose to 32% in 2005–2009.

**Figure 5:**
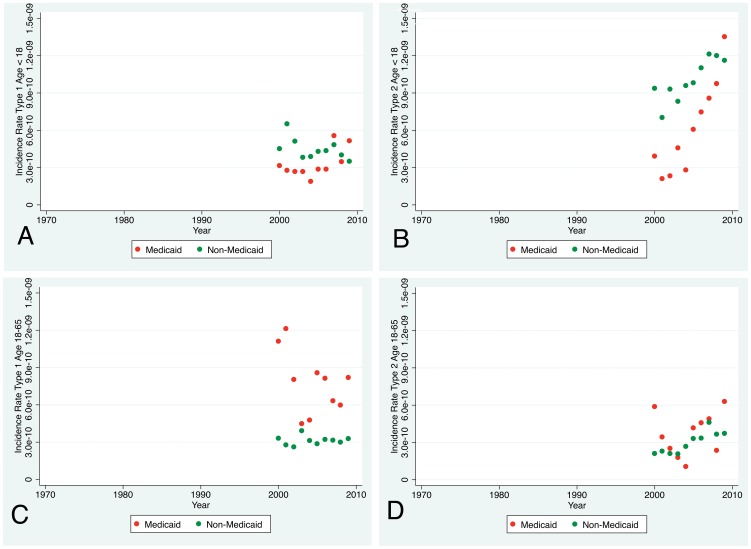
Incidence Rate of Medicaid Coverage. Panel A – The incidence rate of standard (Type 1) admissions of children covered by Medicaid was similar to those covered by other payers. Panel B – The incidence rate of short-stay (Type 2) admissions of children covered by Medicaid was lower than for other payers but rose to similar levels. Panel C – The incidence rate of standard (Type 1) admissions of adults covered by Medicaid was higher than for those covered by other payers. Panel D – The incidence rate of short-stay (Type 2) admissions of adults covered by Medicaid was similar to those covered by other payers. (WA State data of Medicaid eligibility was not available prior to 2000. Incidence rates are small so are expressed in scientific notation. WA State segregates children into ≤18 so the age breakdown is slightly different from the remainder of the manuscript.).

However, the incidence rate of children admitted to the Burn Center with Medicaid coverage was less than for those with other coverage for both standard (Type 1) and short-stay (Type 2) admissions (Fig. 5).

#### Adults

For adults with standard and short-stay admissions, Medicaid coverage increased to 40% in the early 1990s and then declined (Table 2). Commercial insurance coverage followed the reverse pattern. The proportion of Labor & Industries coverage declined over time but has changed little since the early 1990s. Similarly the proportion covered by Medicare changed little over time. The current distribution (2005–2009) is ∼15% Medicare, ∼15% Medicaid, ∼30% Insurance, ∼20% Labor & Industries and ∼20% uninsured on admission.

The ABA-NBR figures for Medicaid coverage of standard and short-stay admissions of adults did not follow this pattern, but are for 1995–1999 11%, 2000–2004 9% and 2005–2009 10%.

The incidence rate of Medicaid coverage for standard admissions is greater than for non-Medicaid coverage (Fig. 5). There was little difference for short-stay admissions.

#### By zip code of residence

King County is the location of Seattle and the Burn Center. The proportion of WA State residents residing in King County and the proportion of WA State admissions from King County are shown in File S4. In summary, the percentage of WA State residents residing in King County decreased from 33% in 1974 to 29% in 2009 and the proportion of WA State admissions from King County declined from over 70% in the late 1970s to ∼40% in 2009. The distribution of payer status on admission from King County did not differ substantially from other WA counties except perhaps for Medicaid coverage of short-stay admissions in the 1980s and early 1990s (Table 3). The distribution of payer on admission for admissions from outside WA State generally included fewer persons with Medicaid coverage and more with commercial insurance (Table 4).

**Table 3:** Comparison of Payer Status on Admission by WA Counties.

Standard	King County	Other	King County	Other	King County	Other
	Mcare		Mcaid		ComIns	
1975–1979	9.8%	6.6%	18.8%	14.2%	24.8%	33.1%
1980–1984	10.1%	6.3%	28.6%	22.6%	35.6%	36.0%
1985–1989	9.1%	9.2%	34.4%	27.5%	26.4%	30.0%
1990–1994	9.7%	7.8%	44.7%	44.0%	25.8%	24.2%
1995–1999	13.6%	12.1%	28.4%	27.3%	31.1%	28.4%
2000–2004	13.9%	12.3%	16.8%	22.4%	30.9%	30.7%
2005–2009	14.9%	15.7%	25.0%	23.9%	28.4%	25.6%
**Short-stay**	**King County**	**Other**	**King County**	**Other**	**King County**	**Other**
	**Mcare**		**Mcaid**		**ComIns**	
1975–1979	1.9%	8.3%	19.4%	16.7%	25.9%	29.2%
1980–1984	3.7%	0.0%	**36.3%**	**17.8%**	31.1%	51.1%
1985–1989	2.6%	4.6%	**31.1%**	**18.4%**	34.0%	40.2%
1990–1994	3.3%	3.7%	**42.9%**	**32.1%**	34.7%	36.6%
1995–1999	5.0%	3.4%	30.7%	26.2%	37.6%	39.2%
2000–2004	8.0%	3.5%	16.5%	16.1%	48.0%	42.3%
2005–2009	5.3%	4.6%	26.7%	23.1%	40.2%	36.3%

There was little difference in payer on admission between King County and other WA counties except for Medicaid coverage of short-stay admissions in the 1980s and early 1990s (bold). (Mcare = Medicare, Mcaid = Medicaid, ComIns = Commercial Insurance).

**Table 4:** Comparison of Payer on Admission by WA versus Out of State.

	Medicare		Medicaid		Insurance	
	WA	Other	WA	Other	WA	Other
1975–1979			**17.4%**	5.7%	5.7%	**20.8%**
1980–1984			**27.7%**	16.5%	16.5%	**46.8%**
1985–1989	7.8%	10.7%	**31.0%**	25.0%	25.0%	**35.7%**
1990–1994	7.4%	13.4%	**43.2%**	34.6%	34.6%	**29.1%**
1995–1999	9.4%	9.9%	**28.2%**	23.8%	23.8%	**26.5%**
2000–2004	9.5%	9.1%	**18.6%**	17.4%	17.4%	**28.3%**
2005–2009	9.6%	8.0%	**24.5%**	20.1%	20.1%	**28.8%**

The proportion of Medicaid coverage was generally less for persons from outside WA State and coverage by commercial insurance greater (bold).

#### Comment on payer status on admission

There continues to be disparity in the income status of persons with burn injuries both in developed and developing countries. Mistri [43] demonstrated economic disparities in burn admissions in New Zealand. Ahuja [44] did likewise in India and van Niekerk [45] in Capetown and Park [46] and Edelman [47] in the United States.

### Site of Event

The majority of injuries in children with both standard and short-stay injuries (81 and 90%) occurred in the home; this did not change over time. The majority of adults with standard and short-stay admissions were also injured at home although the proportion is less than in children, 53 and 71%. Again, there was no trend over time.

Considering persons age 16–65 from WA State, the proportion injured at work declined from 30% in 1975–1979 to 15% in 2005–2009; the incidence rate of work-related burn injuries in persons age 16–65 also declined from 1.8 in 1975–1979 to 1.3 in 1995–1999 and has been stable since then. When these injuries are segregated into type of injury, the absolute numbers changed little except for electrical injuries, which declined over time. The incidence rates of all declined but the majority of the decline occurred prior to year 2000 (Table 5). Mandelcorn [48] also found no reduction in work-related injuries during the past 10 years.

**Table 5:** Numbers and Incidence Rates of Work-Related Admissions.

Year	Flame	Incidence Rate	Scald	Incidence Rate
1975–1979	40	0.34	46	0.39
1980–1984	55	0.39	44	0.31
1985–1989	61	0.41	75	0.50
1990–1994	48	0.29	41	0.25
1995–1999	60	0.32	33	0.18
2000–2004	44	0.22	47	0.23
2005–2009	59	0.27	43	0.20
	**Electrical**	**Incidence Rate**	**Flash**	**Incidence Rate**
1975–1979	39	0.34	42	0.36
1980–1984	34	0.24	43	0.31
1985–1989	50	0.33	52	0.35
1990–1994	51	0.31	50	0.30
1995–1999	39	0.21	39	0.21
2000–2004	21	0.10	53	0.26
2005–2009	26	0.12	53	0.24

The absolute numbers changed little except for electrical injuries that declined over time until approximately year 2000. The incidence rates of all declined but the majority of the decline occurred prior to year 2000.

A similar decline in the proportion of work-related injuries exists in the ABA-NBR data. In the Overall Set for standard and short-stay injuries in adults, there were 27% work-related injuries in 1990–1994, 27% 1995–1999, 21% 2000–2004, and 17% 2005–2009.

#### Comment on site of event

The majority of injuries occur at home, unchanged over time. The absolute numbers of electrical injuries at Harborview declined until year 2000. The incidence rate of work-related burn injuries in persons age 16–65 in WA State declined until approximately year 2000 but has been stable since then suggesting no further improvement. Furthermore there was a decline in work-related injuries in the ABA-NBR.

### Method of Transport

For standard admissions, the use of air transportation increased considerably until the early 1990s when it stabilized at ∼35% of admissions; the median TBSA% transported declined to ∼15% (Table 6). The use of ground transport emergency medical services declined to ∼50% of admissions and the TBSA% of those transported by ground declined to <10% (Table 6). Self-transport also declined to ∼10% (Table 6).

**Table 6:** Method of Transport.

Standard Admissions	Air Ambulance	Median TBSA%	Ground Ambulance	Median TBSA%	Self	Median TBSA%
1975–1979	5%	39%	66%	14%	28%	3%
1980–1984	9%	24%	52%	10%	35%	3%
1985–1989	23%	21%	42%	8%	34%	3%
1990–1994	31%	14%	41%	7%	26%	3%
1995–1999	34%	14%	50%	6%	14%	4%
2000–2004	39%	15%	50%	9%	11%	3%
2005–2009	34%	13%	54%	8%	12%	4%
**Short-stay Admissions**	**Air Ambulance**	**Median TBSA%**	**Ground Ambulance**	**Median TBSA%**	**Self**	**Median TBSA%**
1975–1979	1%	14%	38%	3%	61%	2%
1980–1984	1%	3%	39%	3%	58%	2%
1985–1989	3%	4%	33%	3%	64%	2%
1990–1994	11%	3%	47%	3%	42%	2%
1995–1999	10%	4%	52%	3%	38%	2%
2000–2004	12%	4%	57%	4%	31%	2%
2005–2009	9%	4%	55%	3%	35%	2%

For standard admissions, the use of airlift increased considerably until the mid-1990s and the median TBSA% transported decreased. The use of ground and self-transport declined, as did the TBSA% transported. The pattern was different for short-stay admissions where air and ground transport increased but self-transport declined.

For short-stay admissions, air transport increased to ∼10% of admissions and TBSA% transported was stable at ∼5%. The use of ground ambulance transport increased from ∼40% to ∼55% of admissions for injuries of <5%. Self-transport of short-stay injuries declined from ∼60% to ∼35% for injuries <5%.

#### Comment on method of transport

The use of air transport increased over time and is now utilized for 30–40% of standard admissions and ∼10% of short-stay. Air transport is now used for relatively small injuries, 10–15% TBSA% for standard injuries and ∼5% TBSA% for short-stay. It seems prudent to find alternatives to air transport for short-stay admissions, particularly since the cost of air transport may not be justified for the time savings involved in small burns reaching definitive care. We note that the Harborview catchment area is large and contains expanses of water and mountains so ground transport is not always efficient. This situation is not unique to Harborview as other burn centers also serve large catchment areas [49] and long distance air travel is safe [50].

### Etiology

Flash and flame injuries may be similar in biology but have been segregated historically and were so in the Harborview database; therefore they were separated in this analysis.

**Figure 6:**
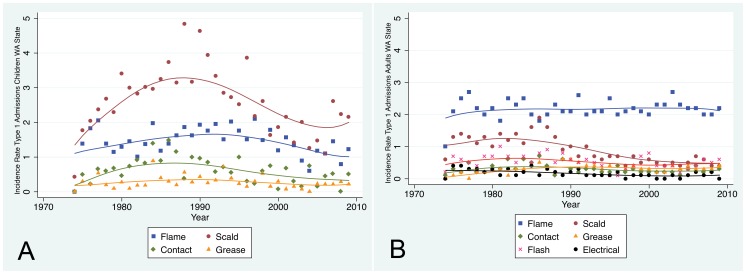
Incidence Rate by Etiology for Standard (Type 1) Admissions. Panel A – The incidence rates for children generally rose until ∼1990, declined until ∼2000 and may have stabilized. The increase and decrease was most profound for scald burns. Panel B – The incidence rates for adults of flame, contact, flash, grease, and electrical burns were largely stable over the years. The incidence rate of scald burns declined in the 1990s and has stabilized since then.

For standard admissions (Type 1) of children, the current proportions are flame ∼30%, scald ∼50%, contact ∼10%, grease ∼5% and flash ∼5%; there were no large changes over time. The incidence rates for children generally rose for all mechanisms until ∼1990, declined until ∼2000 and may have stabilized. The increase and decrease was most profound for scald burns (Fig. 6, Panel A).

For short-stay admissions of children, the current proportions are flame ∼10%, scald ∼50%, contact ∼30%, grease ∼5%, and flash ∼5%, representing a shift from flame injuries in standard admissions to scald and contact in short-stay admissions.

For standard admissions (Type 1) of adults, there was an increase in the proportion of flame burns and decrease in the proportion of scald injuries; the current proportions are ∼50% flame, ∼10% scald, ∼10% contact, ∼10% grease, and ∼15% flash. The incidence rates for adults of flame, contact, flash, grease, and electrical (including both work and not work-related) burns were largely stable over the years (Fig. 6, Panel B). The incidence rate of scald burns declined in the 1990s and has stabilized since then.

For short-stay admissions of adults, the current proportions are flame ∼30%, scald ∼20%, contact ∼10%, grease ∼15% and flash ∼20%, again a shift from flame to scald injuries compared to standard admissions.

In the Overall Set of the ABA-NBR, there was no decline in the proportion of scald injuries for standard (Type 1) admissions of adults.

#### Comment on etiology

In children, scald burns had the highest incidence rate overall but the incidence rate of scald, flame and contact all began to decline in the late 1980s or early 1990s. In adults, flame burns had the highest incidence rate and did not decline as did scald and flash. For both children and adults, the incidence rate of scald injuries declined after about 1990. This would support a conclusion that the tap water legislation of 1980–1984 was effective in preventing accidental scald injuries in adults as reported by Erdmann [51]. However this database does not divide scald injuries into tap water and other types of scald injuries. Peck reviewed tap water injuries and found that they make up only 12–14% of scald injuries [52].

### Body Part Injured

The involvement of any single body part decreased along with the declining TBSA%. However the distribution of body parts injured did not change over time. At present, for standard injuries for all ages the distribution is ∼50% with involvement of the head and neck, arm, hand, and trunk, ∼40% with involvement of the leg and ∼20% for the foot.

### Inhalation Injury

Inhalation injury was a clinical diagnosis made by the treating physicians based on various factors including history of enclosed space fire, estimated/measured carbon monoxide levels >10% in the fire, carbonaceous sputum, and in some instances, PaO2/FiO_2_<400. Not all findings were required to make the diagnosis. Fiberoptic bronchoscopy was rarely performed prior to 2008.

There was no trend in the proportion of admissions with smoke inhalation in conjunction with cutaneous injury for either children or adults, with a mean of 4% of the standard admissions for children and 10% for the adults over time. Similarly there was no trend in the incidence rate, with a mean of 0.2 for children and 0.5 for adults.

There was likewise no trend over time (1995–2009) in the ABA-NBR but the proportions were slightly higher at 5% and 14%. Only 702/5978 (12%) of persons with burn injury of the head and neck also sustained inhalation injury as defined.

### Burn Surgery

**Figure 7:**
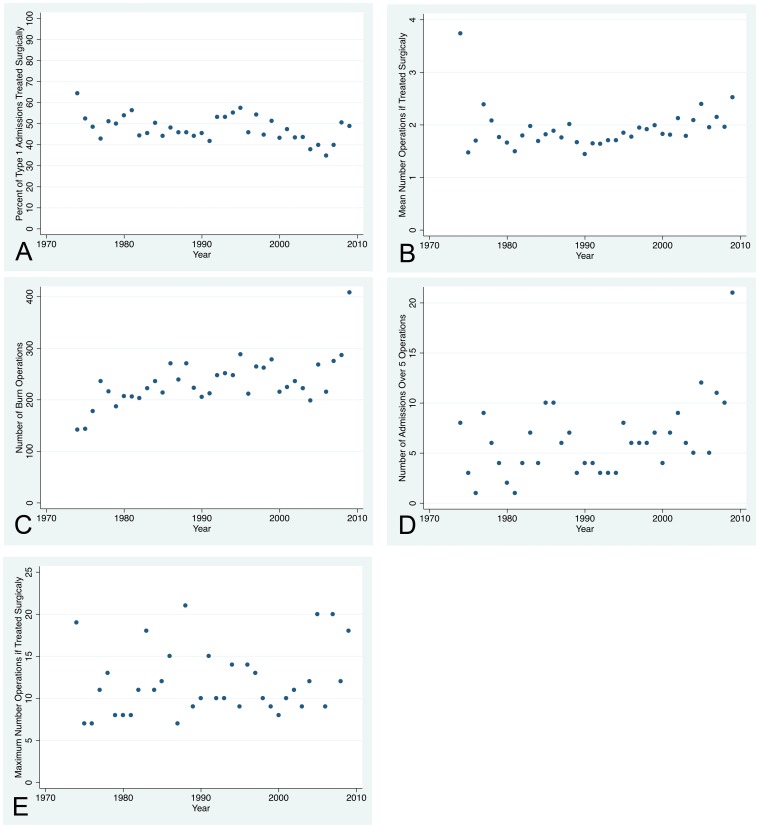
Burn Surgery. Panel A – The percent of standard (Type 1) admissions treated surgically was ∼50% over the entire time period. Panel B – If treated surgically, the mean number of procedures was approximately two for all years, except for the partial year 1974. Panel C – The total number of burn procedures rose when excision became standard procedure and then varied between 200 and 300 until 2009 when it surged to 400. Panel D – Twenty-one persons underwent more than 5 procedures in 2009, which explains the increase to 400 burn procedures. Panel E – The maximum number of procedures for any one person was twenty-one in 1988.

The percent of standard admissions (Type 1) treated surgically was ∼50% over the entire time period (Fig. 7, Panel A). If treated surgically, the mean number of procedures was approximately two for all years, except the partial year 1974 (Fig. 7, Panel B). The total number of burn procedures rose when excision became standard procedure and then varied between 200 and 300 per year until 2009 when it surged to 400 (Fig. 7, Panel C). Twenty-four persons underwent five or more procedures in 2009 compared to 14 in 2008 (Fig. 7, Panel D); the application of Integra® increased from 13 in 2008 to 28 in 2009; and the application of allograft increased from 28 in 2008 to 102 in 2009. These changes likely explain the increase number of burn procedures. The maximum number of procedures for any one person was 21 in which occurred in 1988 (Fig. 7, Panel E).

In the Overall Set of the ABA-NBR, in 2000–2004, 44% of standard admission records indicated one or more procedures were done with a mean of 2.3. In 2005–2009 64% of records indicated one or more procedures were performed with a mean of 3.4.

#### Comment on burn surgery

Approximately 50% of Harborview admissions were treated surgically with two procedures. The number of burn procedures was stable between 200 and 300 per year until 2009 when the use of Integra® and allograft increased.

### Length of Stay for Standard (Type 1) Admissions

Unadjusted median length of stay at Harborview for survivors of standard admissions declined from 17 days in 1975–1979 to 11 in 1985–1989, was then stable for two decades, and then declined further to 9 days in 2005–2009 (Table 7). There was a similar decline in the length of stay in the ABA-NBR data from 11 days in 1995–1999 to 9 days in 2005–2009 (Table 7). To examine factors associated with length of stay, we used age groups ≤5, 6–15, 16–45, 46–65 and >65 years as modified from Moreau [53] and TBSA% groups ≤20%, 21–40%, 41–60% and >60% as modified from Galeiras [54]. This analysis was limited to those persons with standard admissions who survived to discharge (File S5).

**Table 7:** Unadjusted Median Length of Stay for Those Persons Who Survived Standard Admission.

	Harborview		ABA-NBR	
Year Group	Admissions	Length of Stay	Admissions	Length of Stay
1975–1979	897	17.0		
**1980**–**1984**	**1155**	**14.0**		
**1985**–**1989**	**1368**	**11.0**		
1990–1994	1363	12.0		
1995–1999	1260	12.0	19140	11.0
2000–2004	1227	11.0	22383	10.0
2005–2009	1454	9.0	31043	9.0

Unadjusted median length of stay declined at Harborview to 1985–1989, was stable for two decades and then declined again. The declines in 1980–1984 and 1985–1989 (bold) achieved statistical significance in the regression model. Unadjusted median length of stay declined over time in the ABA-NBR data.

Adjusting for other variables, age, TBSA%, and inhalation injury were associated with increased length of stay (p<0.001). Compared to patients 5 years and younger and adjusted for all other variables, the median length of stay was 4.2 days higher for patients aged 46–65 years (95%CI 3.3, 5.1) and 6.6 days higher for patients older than 65 (95%CI 5.1, 8.1). Compared to patients with TBSA% ≤20% and adjusted for all other variables, patients with TBSA% 21–40% had median length of stay 21.1 days longer (95%CI 20.2, 22.0), TBSA% 41–60% had median length of stay 42 days longer (95%CI 40.3, 43.7) and TBSA% >60% 73.0 days longer (95%CI 70.1, 75.7). Inhalation injury was associated with a median length of stay 10.4 days longer (95%CI 9.2, 11.5).

Payer status on admission, residence and time period were all associated with increased length of stay (p<0.001). Medicare and Medicaid were associated with a higher median length of stay compared to commercial insurance coverage (Medicare 3.0(95%CI 1.8, 4.2), Medicaid 0.6(95%CI 0.02, 1.3). Self-pay was not associated with increased length of stay although the number of such persons is small. Residence in WA State counties other than King County (location of Harborview), Alaska, Idaho and Montana were associated with increased length of stay (WA State counties other than King 2.3 (95%CI 2.1, 3.3), Alaska 8.7 (95%CI 7.1, 10.4), Idaho 6.6 (95%CI 4.8, 8.5), and Montana 6.0 (95%CI 4.3, 7.7)). Time period was significantly associated with progressively decreasing length of stay, −1.8 days (95%CI −3.0, −0.7) in 1980–1984 to −7.8 days (95%CI −9.0, −6.7) in 2005–2009.

Johnson [55] reported that one day per percent TBSA remains a good “rule of thumb” to evaluate burn care. At Harborview (Table 8) the mean length of stay/TBSA% for children with standard admissions, TBSA% <20 and residing in King Co. declined over time and was 1.7 in 2005–2009. For adults, the mean length of stay/TBSA% also declined over time and was 3.1 in 2005–2009. For children and adults with TBSA% ≥20 the mean length of stay/TBSA% was slightly greater than one throughout the 36 years. There was little difference between those residing in King County and those residing further away.

**Table 8:** Mean Length of Stay/TBSA% for Survivors of Standard Admissions.

Harborview	TBSA% <20		TBSA% ≥20	
Children	King Co	Outside King Co	King Co	Outside King Co
1975–1979	3.0	3.3	1.3	1.4
1980–1984	3.5	2.7	1.1	1.2
1985–1989	2.8	3.8	0.8	1.4
1990–1994	2.5	2.2	1.4	1.3
1995–1999	1.6	2.1	1.1	1.2
2000–2004	1.5	1.7	1.7	1.3
2005–2009	1.7	1.8	1.1	1.4
**Harborview**	**TBSA% <20**		**TBSA% ≥20**	
**Adults**	**King Co**	**Outside King Co**	**King Co**	**Outside King Co**
1975–1979	5.6	6.3	1.5	1.6
1980–1984	4.4	3.4	1.4	1.5
1985–1989	4.3	3.6	1.3	1.4
1990–1994	3.7	3.4	1.3	1.3
1995–1999	3.8	2.6	1.3	1.3
2000–2004	2.7	2.2	1.3	1.4
2005–2009	3.1	2.3	1.3	1.2
**ABA-NBR**				
**Children**	**TBSA% <20%**	**TBSA% ≥20%**		
1995–1999	2.3	0.9		
2000–2004	2.0	1.0		
2005–2009	2.2	1.0		
				
**ABA-NBR**				
**Adults**				
1995–1999	3.0	1.0		
2000–2004	2.9	1.1		
2005–2009	3.2	1.2		

At Harborview the mean length of stay/TBSA% for children with standard admissions, TBSA% <20 and residing in King Co. declined over time and was 1.7 in 2005–2009. For adults, the mean length of stay/TBSA% also declined over time and 3.1 in 2005–2009. For children and adults with TBSA% ≥20 the mean length of stay/TBSA% was slightly greater than one throughout the 36 years. There was little difference between those residing in King County and those residing further away. There was no change in the ABA-NBR data over time and for TBSA% <20 the ratio was 2.2 for children in 2005–2009 and 3.2 for adults. For persons with TBSA% ≥20 the ratios approximated one.

Using the Length of Stay Set of the ABA-NBR (Table 8) and those persons who survived standard admissions, there was no change over time and for TBSA% <20 the ratio was 2.2 for children in 2005–2009 and 3.2 for adults. For persons with TBSA% ≥20 the ratios approximated one.

#### Comment on length of stay

As length of stay declined for smaller injuries, it is possible that the number of readmissions for infection, skin grafting, and inpatient occupational and physical therapy increased. This detailed data is not available in either the Harborview database or the ABA-NBR. However, data is available in the Harborview database for the period 1994–2002. The number of readmissions ranged from 9–24 with no trend over time suggesting that the number of readmissions did not increase as length of stay decreased. Similarly it is not possible to know precisely from these databases exactly why length of stay declined, although excision of burn injuries and expanded outpatient care of many medical conditions are commonly proposed.

There was no decrease in length of stay/TBSA% for injuries with TBSA ≥20%. However for injuries <20% the ratio declined from 1975 to 1989; this decline could be associated with the increased application of excision and grafting. It seems, however, that early excision did not decrease length of stay for the larger injuries and that length of stay has changed little since ∼1990.

Johnson [55] concluded that “anticipating patient LOS to be 1 day for every TBSA% is still a useful exercise”. We cannot actually compare data since the authors did not publish which ABA-NBR records were used nor did they exclude short-stay admissions, but these data suggest that that criterion may be too stringent and sets a goal that may not be realistic for standard admissions. Of the sixty-eight time points listed in Table 7, only two times was the value of one day/TBSA% achieved. A more realistic goal for standard admissions might be <2 days/TBSA% for injuries ≥20 TBSA% and <3 days/TBSA% for injuries <20 TBSA%.

It is intriguing that the regression indicated that location of residence was associated with increased length of stay whereas the length of stay/TBSA% data did not. This may be because length of stay/TBSA% does not adjust for age, inhalation injury, location of residence, etc.

### Disposition of Persons Surviving

The majority of persons were discharged to home, ranging from 91–100% for children and 82–98% for adults; there was no trend over time. Other discharge options used infrequently include AMA (Against Medical Advice), Other Home, Transitional, e.g. Nursing Home, Extended Care, Other Acute Care, Outside Rehabilitation, Institution, e.g. Jail, Alcohol or Drug Rehabilitation, Shelter, and Street.

Persons requiring inpatient rehabilitation were transferred to the rehabilitation services at Harborview (adults) or Seattle Children’s Hospital (children); this increased from the early years and from 2005–2009 averaged eight persons per year (range 4–12) to the Department of Rehabilitation at Harborview and one per year (range 0–2) to Seattle Children’s Hospital.

### Fluid Resuscitation

Total fluids administered during the first 24-hours increased over time from approximately 4 cc/kg/%TBSA% in 1974–1976 to nearly 6 cc/kg/%TBSA% in 2002–2006 (Table 9). The percent of the first eight-hour fluids delivered prior to admission to the Burn Center increased from ∼50% to ∼80%. Fluids administered in the Burn Center increased from 3.2 to 4.3 cc/kg/TBSA%. Urine volume averaged over the first 24-hours did not change over time, ranging from 0.9 to 1.2 cc/kg/hr.

**Table 9:** First 24 Hour Resuscitation Fluids/Urine Over Time (mean).

	n	Total Fluids	Pre-hospital Fluids/First 8 Hour Fluids	Fluids in House	Urine Output
		cc/kg/%		cc/kg/%	cc/kg/hr
1974–1976	15	4.1	0.5	3.2	1.0
1977–1981	19	4.6	0.5	3.6	1.2
1982–1986	17	4.6	0.6	3.5	1.0
1987–1991	15	4.9	0.6	3.7	1.0
1992–1996	16	5.9	0.7	4.5	1.0
1997–2001	17	5.3	0.7	3.8	1.1
2002–2006	15	5.7	0.8	4.3	0.9

Total fluids increased over time from approximately 4 cc/kg/% in 1974.1976 to approximately 6 cc/kg/% in 2002.2006. The fraction of the first eight-hour fluids delivered prior to admission to the Burn Center increased from 0.5 to 0.8. Fluids administered in-house increased from 3.2 to 4.3 cc/kg/%. Urine volume did not change over time ranging from 0.9 to 1.2 cc/kg/hr.

The unadjusted data (Table 10) suggest increased use of prophylactic tracheal intubation, placement of arterial lines and pulmonary artery catheters, and administration of opioids and decreasing use of central venous pressure lines and colloid may be related to fluids in excess of the Baxter formula during the first 24 hours. To examine the adjusted data, we conducted a regression examining opioid equivalents/24 hrs, benzodiazepine administration, tracheal intubation; central venous pressure and arterial lines; pulmonary arterial catheters; and colloid administration (File S6).

**Table 10:** Utilization of Tracheal Intubation, Lines, Colloid and Opioid Equivalents.

	n	Intubation	CVP	Arterial Lines	PA Catheter	Colloid Administration	Opioid Equivalents (mean)
1974–1976	15	0	2	0	0	4	3.6
1977–1981	19	1	8	4	3	2	4.8
1982–1987	17	8	4	3	0	9	5.2
1988–1991	15	4	2	2	0	5	6.3
1992–1997	16	8	3	6	2	2	7.1
1998–2001	17	10	2	4	10	3	22.7
2002–2006	15	10	2	8	9	3	13.9

The data suggest increased use of tracheal intubation, arterial lines, and pulmonary artery catheters and a decline in the administration of colloid. The administration of opioids steadily increased and then surged in the late 1990s. (n refers to the number of charts studied during the time period. Colloid refers to the number of persons to whom colloid was administered).

An increase of one opioid equivalent was associated with a 0.033 cc/kg/TBSA% increase in first 24-hour fluids (95% CI (0.01, 0.06). In 1997–2001 the opioid equivalents increased 19.1 over 1974–1979 and 10.3 in 2002–2006 (Table 10), translating to an increase of 0.63 and 0.34 cc/kg/TBSA% respectively. Tracheal intubation was associated with a 1.0 cc/kg/TBSA% increase in first 24-hour fluids (95% CI (0.12, 1.88). Use of central venous pressure lines, arterial lines, pulmonary artery catheters, and colloid were not associated with amount of resuscitation fluids administered.

#### Comment on fluid resuscitation and “fluid creep [12]”

“Fluid creep” refers to the increasing administration of fluids in excess of the Baxter formula over the decades. Fluid resuscitation in excess of the Baxter formula was reported in 1996 [56]. There appears to be consensus that supraBaxter fluid resuscitation is associated with complications [57]. But fifteen years later, the cause and prevention of “fluid creep” [12] are still unclear [58] and unresolved [59]. We therefore reviewed fluid therapy over the 36 years in a subset of persons in an attempt to answer the questions 1) was “fluid creep” present in successful resuscitation of uncomplicated injuries, and if so, 2) approximately what year did the phenomenon begin, and finally, 3) what therapeutic events are associated with “fluid creep”?”.

Although a retrospective study cannot definitively answer these questions, it seems that the process is present in resuscitation of uncomplicated injuries, began in the late 1980s or early 1990s, and is not associated with increased urine volumes. Furthermore the data support the notion that the etiology is multifactorial including increasing pre-hospital fluids, pre-hospital prophylactic tracheal intubation, in-hospital fluids, opioid administration and perhaps (although not supported by the regression) increasing use of arterial lines and decreasing use of colloid. It seems that the correction must also be multifactorial and searching for a single cause will be futile.

Alvarado [60] and later Chung [61] have recommended a new formula, “The Rule of 10”, but also mention that the old rules are often misapplied. It seems unlikely that simply swapping rules will solve the issue. Eastman [62] reported that ∼40% of persons with tracheal intubation in the field were extubated in 1–2 days. Since tracheal intubation is associated with increased fluids, this is another issue that might be addressed. Sullivan [63] and Wibbenmeyer [64] also suggested that large doses of opioids might be involved. The extreme rise in opioid administration in 1998–2002 likely followed the events described by Peck [65]. Lawrence [66] uses a different definition of “fluid creep” than Pruitt [12]. Lawrence refers to the increasing fluids requirement of a single individual whereas Pruitt referred to the increasing requirement of populations over time. Perhaps the exact definition does not matter; Lawrence reported that colloid administration rapidly reduces hourly fluid requirements. Blumetti [67] reported that urine output is the important parameter. However in our data urine volumes were stable at approximately 1 cc/kg/hr as fluids administered increased. This suggests that urine output is too far downstream to monitor fluid input, at least after other events have occurred, i.e. excessive administration by first responders, prophylactic tracheal intubation, etc. With the other variables in play, it might be a “deadly pitfall” as suggested by Hartford [68]. Again, it appears that a multifactorial response will be required.

It is possible that with a one page, prospective data collection protocol and 10 centers with sufficient admissions, in one year’s time the data would be sufficient and likely clear. In 1999, the U.S. Department of Veterans Affairs (VA) launched an initiative called “Pain as a Fifth Vital Sign”. If pain is the 5^th^ vital sign, perhaps the first 24-hour fluids should be the 6^th^ vital sign in burn-injured persons. The data collection form might be similar to the document described by Chung [69] for use in the military. These forms might be required for verification and be stored in the ABA-NBR.

### Hospital Mortality

Persons placed on comfort care (see the following section on Comfort Care) immediately upon arrival were included in mortality analyses because our goal is to study overall burn mortality, not treatment efficacy. Unadjusted case fatality at Harborview declined from ∼12% in 1974–1979 to ∼6% in 1985–1994, rose to ∼8% in 1995–2004, and declined to ∼6% in 2005–2009 (Table 11). Case fatality with the Overall Set of the ABA-NBR and standard admissions declined from 6.9% 1995–1999 to 6.0% 2005–2009 (Table 11).

**Table 11:** Unadjusted Mortality.

	Harborview		ABA-NBR	
Year Group	Standard Admissions	Mortality	Standard Admissions	Mortality
1974–1979	1081	11.9%		
1980–1984	1258	8.2%		
1985–1989	1453	5.8%		
**1990**–**1994**	**1445**	**5.7%**		
1995–1999	1362	7.5%	20649	6.9%
2000–2004	1328	7.6%	24030	6.6%
**2005**–**2009**	**1538**	**5.5%**	31663	6.0%

At Harborview unadjusted mortality declined to the mid-1990s, relapsed 1995–2004, and then returned to the 1985–1989 level. The periods 1990–1994 and 2005–2009 (bold) achieved statistical significance in the regression model suggesting no improvement since 1990. Unadjusted mortality declined from 6.9% to 6.0% in the ABA-NBR data.

#### Regression of mortality over time with Harborview data and standard admissions

Since unadjusted case fatality does not control for age, TBSA%, etc. [70], to further evaluate hospital mortality we performed logistic regression of mortality in standard admissions on age, TBSA%, inhalation injury, sex, race/ethnicity, and time period. To evaluate change over time, we stratified year of admission into 1974–1979 and then five-year time periods thereafter. The number of records by time period ranged from 1,108 in 1974–1979 to 1,539 in 2005–2009 (File S7).

Age, TBSA%, inhalation injury, sex, and time period were all associated with mortality (p<0.001). Adjusting for other variables, the odds of death in age group 6–15 years was less than in children ≤5 years, OR 0.50 (95%CI 0.25, 1.01), as was the odds of death in age group 16–45, OR 0.78 (95%CI 0.49, 1.24). The odds of death were higher for ages 46–65 (OR 4.01; 95%CI 2.51, 6.41) and for patients over 65 (OR 28.09; 95%CI 17.73, 44.50) compared to patients ≤5 years. The odds of death increased steadily with TBSA%. Compared to patients with TBSA% ≤20%, the OR was 11.11 (95%CI 8.09, 15.27) for TBSA% 21–40%, OR 44.39 (95%CI 27.84, 61.52) for TBSA% 41–60%, and OR 444.20 (95%CI 287.85, 685.48) for TBSA% over 60%. The odds of death increased with inhalation injury, OR 6.15 (95%CI 4.65, 8.13). The odds of death also increased with female sex, OR 1.68 (95%CI 1.30, 2.16). There was no evidence that race/ethnicity was associated with mortality.

The previous findings merely confirm many other published observations. In this study examining change over time, adjusting for other variables and compared to 1975–1979, the odds of death were 17% lower in 1980–1984, 33% lower in 1985–1989, and 38% lower in 1990–1994. During the period 1995–1999, the odds of death were similar to that in 1975–79. The smallest odds of death were for the most recent time period, with OR = 0.39 (95%CI 0.25, 0.59) compared to the earliest time period (61% lower odds of death).

We also examined adjusted changes in mortality over time for standard admissions separately for children and the elderly. There was no improvement over time for children ≤5 years and for those 6–15 years. For those persons >65 years (n = 786), there was no improvement over time until 2005–2009 when the odds of death declined, OR 0.32 (95%CI 0.13, 0.77). Among persons with large burns of TBSA% >60% (n = 296), there was no trend toward improvement over time but 1995–1999 was notable with OR 3.6 (95%CI 0.7, 18), compared to 1975–79.

#### Regression of hospital mortality over time in ABA-NBR data standard admissions

We examined mortality using the Mortality Regression Set of ABA-NBR records, adjusting on age, TBSA%, inhalation, sex, race/ethnicity, and time period (File S8).

The ABA-NBR data are consistent with the Harborview data. The only differences are, in the ABA-NBR data, statistical significance of the findings for age group 6–15 (0.47 (95% CI 0.35, 0.65)), age group 16–45 (1.86 (95%CI 1.53, 2.26), and Non-White race/ethnicity (1.21 (95%CI 1.11, 1.33) and the failure of the improved survival in 2005–2009 to achieve statistical significance.

The analysis was repeated for Type 1 admissions of children and the elderly separately for 1995–1999, 2000–2004 and 2005–2009. There was no change over time for ages 6–15 years. For those people ≤5 years, the odds of death increased OR 1.5 (95%CI 1.1, 1.2) between 1995–1999 and 2005–2009. For ages >65 years (n = 9,854) the odds of death declined 2000–2004 OR 0.7 (95%CI 0.6, 0.8) and 2005–2009 OR 0.5 (95%CI 0.45, 0.6). Pham [71] also studied mortality in the elderly in the ABA-NBR and, although the authors did not segregate standard and short-stay admissions, found improved survival. With standard admissions and TBSA% limited to >60% (n = 2,368), unlike the Harborview data, in 2005–2009 there was improved survival compared to 1995–1999 with OR 0.5 (95%CI 0.4, 0.65).

#### rBaux mortality model applied to Harborview and ABA-NBR standard admission data

As described in Methods, we segregated the revised Baux (rBaux) scores into seven groups for this analysis, (1) ≤75, (2) >75 and ≤85, (3) >85 and ≤100, (4) >100 and ≤115, (5) >115 and ≤130, (6) >130 and ≤150 and (7) >150.

Mortality generally declined over time in the Harborview data and in 2005–2009 matched rBaux prediction except for Group 2, which was less than predicted, and Group 5, which was slightly more than predicted (Table 12). Using the Mortality Model Set of ABA-NBR records, there was little change over time and mortality matched the rBaux prediction in all groups as expected since the ABA-NBR data from 2000–2007 was used to build the rBaux system. However the ABA-NBR data from 1995–1999 also matched, suggesting that rBaux scores are robust over time and that there has been little improvement in survival since ∼1995.

**Table 12:** Comparison of the rBaux Model with Harborview (HMC) and ABA-NBR Data for Standard Admissions.

rBaux Group	rBaux Score	rBaux Predicted Mortality	1975–79 Observed Mortality %/n/CC	1980–84	1985–89	1990–94	1995–99	2000–04	2005–09
HMC									
1	≤75	≤5%	1.3%/794	1.0%/1040	0.9%/1233	0.4%/1219	0.9%/1124	1.2%/1080/1	0.6%/1246/1
2	>75≤85	>5% ≤10%	13%/55	9%/57	9%/79	5%/57	9%/71	9%/71/1	**2%/90**
3	>85≤100	>10% ≤26%	35%/71	27%/56	16%/57	19%/78	27%/83/1	21%/85/1	13%/104
4	>100≤115	>26% ≤52%	73%/37	58%/31	55%/31	43%/42	53%/34/4	53%/40/1	33%/43/3
5	>115≤130	>52% ≤78%	81%/32	82%/22	87%/15	80%/20	79%/19/3	71%/28/5	**84%/19/8**
6	>130≤150	>78% ≤94%	100%/19	96%/23	90%/19	92%/12	100%/18/7	92%/13/4	84%/25/2
7	>150	>94%	100%/8	100%/14	100%/10	100%/8	100%/13/10	100%/11/1	100%/11/7
ABA-NBR									
1	≤75	≤5%					0.9%/16475	1.0%/18260	1.1%/24084
2	>75≤85	>5% ≤10%					8%/1294	9%/1444	7%/1995
3	>85≤100	>10% ≤26%					18%/1298	18%/1560	18%/2038
4	>100≤115	>26% ≤52%					43%/682	46%/722	42%/859
5	>115≤130	>52% ≤78%					65%/386	66%/381	66%/473
6	>130≤150	>78% ≤94%					86%/233	89%/257	88%/å328
7	>150	>94%					96%/154	99% 149	99% 211

Mortality generally declined over time in the Harborview standard admission data and in 2005–2009 mortality matched rBaux prediction save in Group 2, less than predicted, and 5, slightly more than predicted (bold). There was little change in the ABA-NBR standard admission mortality over time and mortality matched the rBaux predicted mortality in all groups. Since the ABA-NBR data from 2000–2007 was used to build the rBaux system, this is not surprising. However the ABA-NBR data from 1995–1999 also matched suggesting that rBaux scores are robust over time and that there has been little improvement in survival since 1995. (x%/y/z x = percent observed mortality, y = sample size and CC = the number placed on comfort care).

Comparison to the rBaux model in Table 12 also adds detail to the regression mortality findings. There was improved survival in groups 1, 2, 3, 4, and 6 over time, with the less severe groups exhibiting an increase in mortality in 1995–1999. These results indicate that the improved survival was in the small to medium rBaux scores.

**Figure 8:**
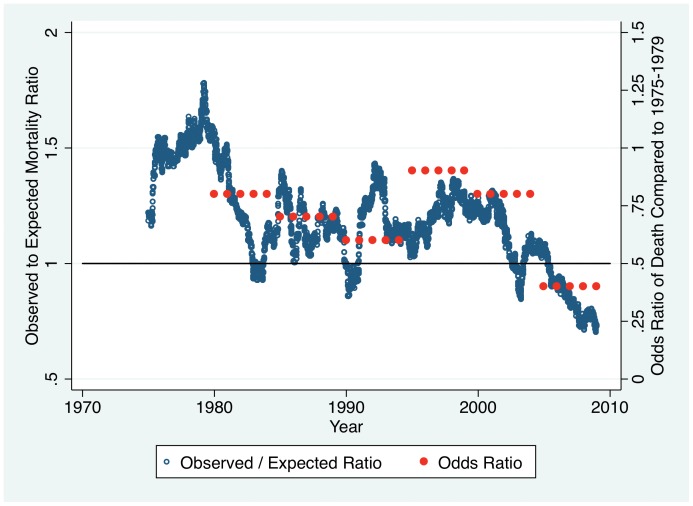
Mortality Standardized to the rBaux Predicted. Observed mortality was about 1.5 times higher than rBaux predicted mortality prior to 1980, and was about 1.25 times rBaux predicted mortality from ∼1980 to ∼2000. Since ∼2005 observed mortality has been less than rBaux predicted mortality. The adjusted odds of death from the regression (shown in red) was higher for all half-decades compared to 1975–1979 except for the most recent half decade.

Further clarity of the regression findings may be seen in a graph of mortality standardized to that predicted by the rBaux model (Fig. 8). To assess trends in the observed/expected ratio over time, we computed a moving average of the observed/expected ratio using a moving window of 2,000 patients, which corresponded to about two years of calendar time. The moving average was created by calculating the observed/expected ratio on a series of different subsets of the full dataset. In our case, we computed the ratio for the first 2,000 patients in the dataset, then for patient #2 and on to patient #2001, and so on. Observed mortality was about 1.5 times higher than rBaux predicted mortality prior to 1980, and was about 1.25 times rBaux predicted mortality from ∼1980 to ∼2000. Since ∼2005 observed mortality has been less than rBaux predicted mortality. The adjusted odds of death was higher for all half-decades compared to 1975–1979 except for the most recent half decade.

To convert this to numbers of persons, with the rBaux model in 1975–1979, 42.8 people out of 1,182 admissions died who now would be expected to survive. In 2005–2009, 18.4 people out of 3,252 admissions survived who were expected to die. This totals to 61.2 per five years who now survive, approximately one per month. Compared to 1985–1989, it is one person every two months.

#### Regression of mortality over time with the rBaux score, Harborview data and standard admissions

After confirming the validity of the rBaux model, we reran the mortality regression using the rBaux score rather than the age, TBSA% and inhalation groups (File S9). The odds of death for the rBaux score were 1.09 (95%CI 1.082, 1.095). This, however, renders the association with age, TBSA% and inhalation injury individually not apparent. The regression confirmed the findings by time period. It differed in that for race/ethnicity the odds of death were 1.59 (95%CI 1.16, 2.17) suggesting that perhaps there is an association with race/ethnicity, as suggested by the ABA-NBR data.

#### Comment on mortality

Unadjusted case fatality declined to 5.7% in 1990–1994, relapsed in 1995–2004, and returned to 5.5% in 2005–2009, i.e. the largest improvement occurred two decades ago, much as reported by Danilla Enei [72], Muller [73] and Jaskille [74]. Nor did we find improvement in the ABA-NBR data since 1995. This suggests that burn care has achieved the floor of survival, as also noted by Blaisdell [75]. The regression revealed improved survival for the elderly as also reported by Macrino [76] Gomez [77] and Lionelli [78] although none of the authors clearly segregated standard and short-stay admissions. The association with gender is clear but the association with race/ethnicity is not. Current Harborview survival statistics match the rBaux model quite well as do ABA-NBR survival statistics for 1995–1999, suggesting that the model will be useful.

### Trajectory to Death

**Figure 9:**
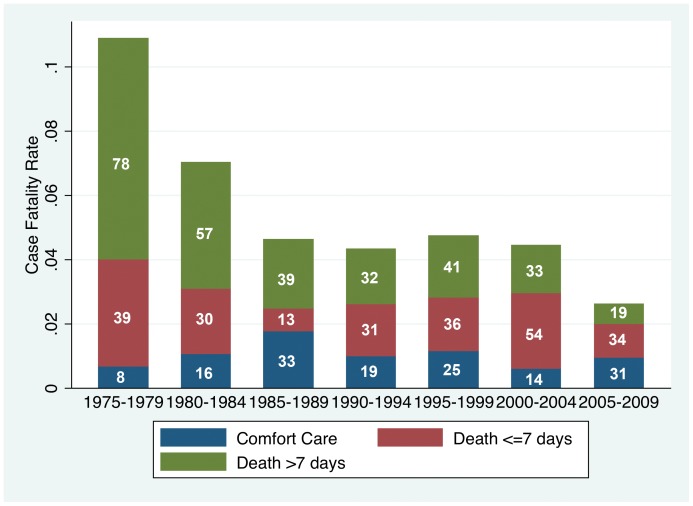
Days to Death by Groups. There was no trend over time to the number of persons placed on Comfort Care or to the number of persons who were not placed on comfort care but who died within eight days. However the number of persons who died after seven days decreased to the mid-1980s and again in 2005–2009.

Days from admission to death declined over the 36 years, going from median (IQR) 10 days (1–16) in 1980–1984 to 1(1–5) in 2005–2009. To explore this we stratified death into three groups: 1) comfort care on admission (all died ≤2 days), 2) death ≤7 days excluding Group 1, and 3) death >7 days (Fig. 9). The case fatality rate of Groups 1 and 2 did not significantly change over time but deaths after seven days (Group 3) declined to the late 1980s, then was stable for twenty years, and then declined again in 2005–2009.

The trajectory to death with the Length Stay set of the ABA-NBR also declined, going from median 7 (IQR 2–15) in 1995–1999 to median 4 (IQR 1–11) in 2005–2009.

It is not possible to compare the three groups above to the ABA-NBR data, as the ABA-NBR data has no reliable field indicating comfort care. However, the case fatality rate of Group 3 was 0.056 in 1995–1999, 0.054 in 2000–2004 and 0.048 in 2005–2009, also a significant change with p = 0.001.

#### Comment on trajectory to death

It is likely that the decline from 1975 to 1985 resulted from the increased application of early excision as reported by Ong [79]. It is not clear however, why late deaths declined from 2000–2004 to 2005–2009 and the trend will need to be re-assessed with 2010–2014 data.

### “Unprecedented Survival” and “Unsurvivable Burn Injury”

One of the parameters sometimes used in predicting whether or not resuscitation is futile is the concept of “unprecedented survival” or “unsurvivable burn injury”. Both concepts are flawed because, even if “unprecedented”, survival might be achieved and “unsurvivable” requires great foresight. Nevertheless, it is important to know what has been achieved when discussing resuscitation plans with injured persons and families.

The highest rBaux scores with survival achieved at Harborview are shown in Table 13. However, the ABA-NBR data with the Mortality Model Set of the ABA-NBR lists survivors with higher rBaux scores, suggesting that survival with higher scores is possible.

**Table 13:** Highest rBaux Scores with Survival Achieved at Harborview and in the ABA-NBR.

Age	Harborview	ABA-NBR
≤5	93	112.8
6–15	89	123.6
16–45	136	152.3
46–65	136	157.3
>65	146	147.4

The highest rBaux Scores achieved at Harborview and in the ABA-NBR.

For this investigation we required that the record be in the Overall Set of the ABA-NBR, that the person be discharged from care in the burn center, and that numbers of operations and length of stay be reasonable, e.g. a record with length of stay of 479 days would not be considered a valid record. The highest rBaux scores we found are shown in Table 13.

It is difficult to determine rBaux scores in published manuscripts as the detailed information by patient is usually not reported. However Lumenta [80] reported a 47 year old with 80% TBSA% and inhalation injury who survived (rBaux = 144) and a 36 year old with 90% TBSA% and inhalation injury who survived (rBaux score = 143).

#### Comment on “unprecedented survival” and “unsurvivable burn injury”

Injuries with rBaux scores in the range of 110 (age ≤5), 120 (age 6–15), 150 (age 16–45), 160 (age 46–65), and 150 (age >65) have survived, and therefore survival of persons with these rBaux scores is not “unprecedented” or “unsurvivable”.

### Comfort Care

**Figure 10:**
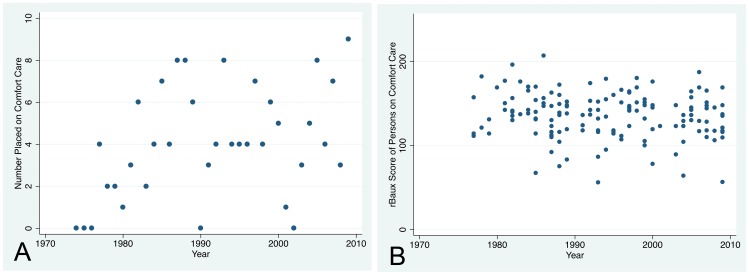
Frequency and rBaux Scores of Comfort Care. Panel A – There was no trend over time to the number of persons placed on comfort care. Panel B – There was no trend over time to the rBaux scores of persons placed on comfort care.

The frequency with which comfort care was selected and the rBaux scores of the persons treated in this fashion are shown in Fig. 10. There was no trend over time in to the number of persons placed on comfort care (Fig. 10, Panel A) or the rBaux scores of those persons (Fig. 10, Panel B).

#### Comment on comfort care

In 2005–2009 there were 84 deaths in the Harborview Burn Center and 31 (37%) were placed on comfort care on arrival (Fig. 9). Hemington-Gorse [81] reported on 32 deaths at St. Andrew’s Centre for Burns in Chelmsford, UK between 1/1/2008 and 12/31/2009. Eleven (34%) were place on comfort care on arrival. The percentages are similar, suggesting that the decisions for comfort care at Harborview may be similar to other centers.

### Summarized & Highlighted Results

Since the Results section is extensive, we have summarized and highlighted the findings.

1. The number of standard admissions has been stable for two decades and the incidence rate declined in the 1990s but has since stabilized. The number of short-stay admissions increased markedly as did the incidence rate; in 2005–2009 short-stay admissions made up>50% of all admissions. The proportion of short-stay admissions increased in the ABA-NBR as well.
2. The number of admissions with TBSA >20% was stable over time; the incidence rate declined to 2005–2009. The median TBSA% and 75^th^ percentile declined to the mid-1980s but no further thereafter. The number of records in the ABA-NBR with TBSA >20% declined over time.
3. The median age increased due to increasing proportions of persons over 45 years of age. Increasing age was also evident in the ABA-NBR.
4. There was disparity in race/ethnicity and payer status.
5. Most injuries occurred at home. The incidence rate of injuries on the job declined to the mid-1990s but no further thereafter. This was also observed in the ABA-NBR.
6. The use of air transport increased, even for injuries of small TBSA%.
7. In children, scald remained the most common etiology and the incidence rate did not decline during the past decade.
8. The incidence rate of inhalation injury did not change over time and affected 4% of standard admissions of children and 10% of adults. Proportions in the ABA-NBR were slightly higher.
9. Fluids in excess of the Baxter formula did occur with uncomplicated resuscitations, was present in the early 1990s, was not associated with increased urine volume, and continued to 2005–2009. The cause may be multifactorial.
10. The number of burn operations was stable over time until it surged in 2009. Approximately 50% of persons with standard admissions were treated surgically with approximately two procedures. In the ABA-NBR, in 2005–2009, the percent of records indicating surgery and the mean number of procedures were higher, 64% and 3.4.
11. Unadjusted length of stay declined over time. Length of stay increased with increasing age, increasing TBSA%, inhalation injury, Medicaid/Medicare payer and zip codes of residence outside of King County. Length of stay of one day per TBSA% was rarely achieved, either at Harborview or in the ABA-NBR, especially for injuries of small TBSA%.
12. Unadjusted case fatality with standard admissions declined from ∼12% to ∼6% until the mid-1980s; there was little decline thereafter. Mortality increased with age, TBSA%, inhalation injury, and female sex throughout the 36 years. The odds of death decreased over time although the absolute number is small. The odds of death improved for persons >65 years. There was no improvement in survival for injuries >60 TBSA%. In the ABA-NBR, there was little improvement in overall survival after 1995–1999; there was however, improved survival for persons >65 years of age and for persons with TBSA% >60.
13. The rBaux model predicted survival quite well.
14. The trajectory of days to death changed with diminished numbers of persons dying after seven days from injury.
15. Survival with rBaux scores in the range of 110 (age ≤5), 120 (age 6–15), 150 (age 16–45), 160 (age 46–65), and 150 (age 65) is not “unprecedented”.
16. There was no trend over time in the use of comfort care at Harborview.

## Discussion

### Use of the ABA-NBR

We found several manuscripts using the ABA-NBR data but none reported which records were studied and how they were selected. The National Burn Repository 2011 Report (Dataset Version 7.0) (http://www.ameriburn.org) likewise does not include the record numbers and the record selection method. This makes comparison very difficult. It would seem helpful for authors using ABA-NBR data to include a file containing the record numbers used in the study as done with File S3. Furthermore it is true that culling the ABA-NBR of irrelevant records is required as noted by Pavlovich [20]. Therefore the methods used to filter records should also be provided as in File S2.

### Implications for the Organization and Delivery of Burn Care at Harborview

The current burn center model emerged in the 1940s and surged in the 1970s [82], [83] to serve the needs of persons that could not be efficiently and properly managed in unspecialized acute care hospitals. It is fundamentally an inpatient model, an intensive care unit with an attached ward, operating room and clinic, staffed primarily by general surgeons; Heimbach described the attributes of the model [35]. For the purpose for which it was designed, the model has been and still is, a success [82].

However our analysis confirms that many changes have occurred at Harborview over the past three decades, some of which have been described in other burn centers. The total number of admissions increased; the number of large burns declined; the number of burn related operative procedures increased; the number of short-stay and other non-operative, non-intensive care unit admissions increased; the length of stay declined; and mortality declined. In addition to these burn changes, the wound care methods that have been found to be beneficial for burn injuries are often applicable to other types of wounds. Therefore as Kastenmeier [36], Imahara [84], and Pham [85] have reported, persons with various other wounds are being referred to and treated at Harborview and other burn centers, further increasing the numbers of persons needing care and making resource allocation even more complex. And finally the Federal requirements for chart documentation have grown enormously. Ten minutes per day per patient with fifteen patients is 2.5 hours/day.

As a result of these changes, the Burn Center is no longer a homogeneous intensive care unit with an attached burn operating room, ward and burn clinic treating injuries of large TBSA%. Rather it is a voluminous operating room, heterogeneous ward, and large clinic with an attached intensive care unit. The old Heimbach/Engrav model may still be required, but is no longer sufficient. In fact, Gamelli called for change in 2006 [86].

What can be done to adapt to the updated set of needs, to the shift from large injuries in the intensive care unit to smaller outpatient injuries? We proffer several ideas for consideration. First, we could simply expand everything, more surgeons, more nurses, more therapists, more outpatient facilities, etc. A second option is to selectively expand outpatient care (medical, nursing, therapy). Third, we could develop satellite burn centers in Montana, Idaho, Eastern Washington, and Alaska to care for the smaller, simpler injuries. Blaisdell [75] has already suggested that burn centers be identified with a “level” designation; these new satellites could be “level 2”. A fourth option is to offload various Burn Center activities to “burn team extenders”, ICU care to intensivists trained in the needs of burn injury, outpatient non-operative care to other physicians trained in non-operative burn management, outpatient occupational and physical therapy to non-Burn Center therapists trained in the needs of burn injury. Finally, we could fully develop “Smartphone Medicine” to treat “simple” injuries over great distances.

Some systems have already been reported elsewhere to address similar needs and situations. Sagraves [87] described burn care provided for short-stay injuries by rural trauma surgeons and a dedicated burn nurse. Burn surgeons provide consultation via email and telephone. The authors reported clinical success with this system, which lessens the burden on the burn center and minimizes patient travel time and family lodging expenses. Blaisdell [75] described “de facto” regionalized care in Maine with no difference in mortality when compared to the ABA-NBR.

### The Proposed Outcome Paradigm Shift

In 1992 Salisbury [88] and 1993 Warden [89] suggested it was time to consider outcomes other than mortality. In 1993 the National Institute on Disability and Rehabilitation Research began to fund burn rehabilitation research. In 2003 Shakespeare [90] called for a paradigm shift away from assessment of injury severity towards quality of outcome. Later Pereira [70] and Jaskille [74] reiterated the call.

The data from Harborview and the ABA-NBR confirms that mortality and length of stay have changed little since the mid-1980s, reinforcing the call for other outcome measures. But still, fifteen years after the first call for change, the Guidelines for the Operation of Burn Centers [91] do not speak to these matters in any detail. Richard and twenty-two other authors [92] described the situation in some detail but suggestions to remedy the situation were sketchy. There are many possibilities but several warrant discussion.

The ABA maintains the ABA-NBR, an acute burn database, and the National Institute on Disability and Rehabilitation Research (NIDRR) Burn Model Systems maintain a burn rehabilitation database, and both are decades old with large numbers of records. However the two are separate and distinct and it is difficult or impossible to study the contents of both simultaneously. It seems timely to mix/match/merge the two databases.

It would also seem timely to rewrite the Guidelines for the Operation of Burn Centers to recognize the shift from mortality to psychological, social, vocational, and functional outcome, even to include the core of current outcomes research, i.e. how people function and their experiences with their care (http://www.ahrq.gov/clinic/outfact.htm).

And finally, the present NIDRR Burn Model System funding ends 9/30/2011 and may or may not be renewed. If it is renewed, a call for comments will likely be published in the Federal Register. It will be important for the burn community to comment in order to shape the structure of the renewal.

### Comparison of Outcome to Known Events

It is known that various changes in burn demographics, prevention, and care occurred at certain times, excision of burn wounds (1977–1979), widespread use of smoke detectors (1980–1984), discontinuing immersion hydrotherapy (early 1980s), tap water legislation (1980–1984), early enteral feeding and minimal use of antibiotics (1985–1989), restrictive transfusion (1990–1994), Integra® (1995–1999), Harborview Transfer Center (1995–1999) and pain declared the 5^th^ Vital Sign (1995–1999). It would be a valuable contribution to use the database to comment on the value of these changes. Unfortunately the vagueness of the timing and the vagueness of the outcome makes this impossible as noted by Gomez [77]. It is true that mortality and length of stay had decreased by 1985–1989 and opioid administration increased markedly in 1995–1999.

### Accuracy of Mortality Models

It is a fair question “can mortality be predicted on admission?” Pereira [70] stated “clinical decisions …using variables obtained at admission are essentially inaccurate”. It is true that the longer one waits, the more accurate the prediction. However the rBaux model, based upon three variables known on admission, is quite accurate.

## Supporting Information

File S1 **Populations WA State.** This file includes the populations of Washington State from 1974 to 2009 by age and race.
(XLS)

File S2 **ABA-NBR Filters.** The ABA-NBR includes many records not relevant to this study. This file includes the filters used to select relevant records.
(DOC)

File S3 **ABA-NBR Records.** This file includes the ABA-NBR record numbers included in each analysis set permitting the reader to reproduce the results.
(CSV)

File S4 **King County.** The percent of Washington State residents living in King County (the location of the Burn Center) and the percent of Washington State admissions to the Burn Center from King County are included in this file.
(XLS)

File S5 **LOS Regression.** This is the STATA quantile regression of length of stay on age, TBSA%, race/ethnicity, gender, inhalation injury, payer and time period.
(DOC)

File S6 **Fluid Regression.** This is the STATA regression of fluids/kg/% on opioid equivalents, benzodiazepines, tracheal intubation, central venous pressure lines, arterial lines, pulmonary artery catheters, and colloid.
(DOC)

File S7 **Mortality Regression Harborview.** This is the STATA regression of mortality with standard admissions at Harborview on age, TBSA%, inhalation injury, gender, race/ethnicity and time period.
(DOC)

File S8 **Mortality Regression ABA-NBR.** This is the STATA regression of mortality with standard admissions in the ABA-NBR on age, TBSA%, inhalation injury, gender, race/ethnicity and time period.
(DOC)

File S9 **Mortality Regression Harborview rBaux.** This is the STATA regress of mortality with standard admissions at Harborview on rBaux score, gender, race/ethnicity and time period.
(DOC)
