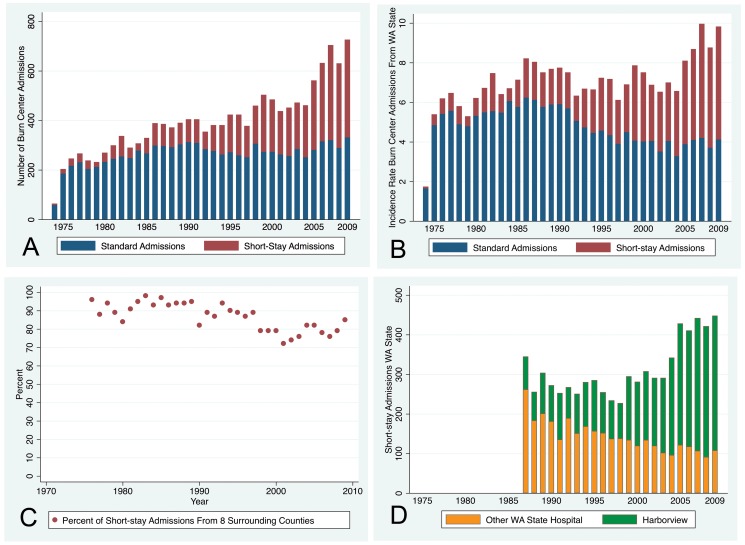# Correction: Harborview Burns – 1974 to 2009

**DOI:** 10.1371/annotation/8dffa635-e876-48e1-958a-0016d3618d64

**Published:** 2013-10-23

**Authors:** Loren H. Engrav, David M. Heimbach, Frederick P. Rivara, Kathleen F. Kerr, Turner Osler, Tam N. Pham, Sam R. Sharar, Peter C. Esselman, Eileen M. Bulger, Gretchen J. Carrougher, Shari Honari, Nicole S. Gibran

Graph B was duplicated as Graph A in Figure 2. Please see the corrected Figure 2 with Graph A here: 

**Figure:**