# Supplementary material for: Harborview Burns – 1974 to 2009
Source: PLoS One. 2012 Jul 5;7(7):e40086. doi: 10.1371/journal.pone.0040086 (PMC3390332; doi:10.1371/journal.pone.0040086)
Supplement: File S2 — ABA-NBR Filters. The ABA-NBR includes many records not relevant to this study. This file includes the filters used to select relevant records. (DOC) [file pone.0040086.s002.doc]

**Supporting File S2 – Filters Used to Select ABA-NBR Records**

To remove University of Washington records

if fac_key = xx; drop

To select admissions for new burn injuries

Step 1

if admstat_cat = valid; retain

Step 2

if admstat_cat = Unknown, if admstat = Initial Admission or Initial Admission, Burn Injury Related; retain

Step 3

if admstat_cat = Unknown, if admtype = Initial Admission; retain

Step 4

if admstat and admtype conflict; drop

To select known etiology

if etiology and etiocode_cat = Burn, Unspecified, Chemical, Contact with Hot Object, Electrical, Fire/Flame, Other Burn, Visible & ultraviolet sources, or Scald; retain

To select known age

if age > 0 and age <= 90; select, if not and man_age > 0 and <= 90; select, if not; drop (in the ABA-NBR ages > 90 are recorded as 90)

To select known TBSA

if areatot > 0 and areatot <= 100; retain

To select known death

the field "dead" is known to be invalid

if hospdisp_cat = Death or Death in Hospital or Died or Arrival; retain

To select survival

if hospdisp_cat <> the death codes above and <> Unknown; retain

To select known gender

if sex = Male or Female; retain

To select known presence of inhalation injury

if inhal_yes = 0 or 1; retain

To select known race/ethnicity

if race = Asian, Black, Hospanic, Native American, Other, White; retain

To select known year of admission

if edinyrx = valid; select, if not and injyr = valid; select, if not and dcyear = valid; select

To select known length of stay

if hospdays > 0 and < 365; keep (arbitrary decision that hospdays > 365 was an entry error)
