# Supplementary material for: Harborview Burns – 1974 to 2009
Source: PLoS One. 2012 Jul 5;7(7):e40086. doi: 10.1371/journal.pone.0040086 (PMC3390332; doi:10.1371/journal.pone.0040086)
Supplement: File S5 — LOS Regression. This is the STATA quantile regression of length of stay on age, TBSA%, race/ethnicity, gender, inhalation injury, payer and time period. (DOC) [file pone.0040086.s005.doc]

Supporting File S5

STATA Regression 1 – Quantile Regression of Length of Stay on Age, TBSA%, Race/Ethnicity, Gender, Inhalation Injury Payer and Time

xi: qreg days1burncenter i.agegrpmodMoreau i.tbsagrpmodGaleiras i.racegrp i.sex i.inhalation1no2yes i.payorforregress i.LOSres i.yrgrp748085etc if racegrp < 9 & sex < 9 & inhalation1no2yes < 9 & (payorforregress < 4 | payorforregress == 7) & LD == 0 & days1burncenter > 2;

i.agegrpmodMo~u _Iagegrpmod_1-5 (naturally coded; _Iagegrpmod_1 omitted)

i.tbsagrpmodG~s _Itbsagrpmo_1-4 (naturally coded; _Itbsagrpmo_1 omitted)

i.racegrp _Iracegrp_1-9 (naturally coded; _Iracegrp_1 omitted)

i.sex _Isex_1-2 (naturally coded; _Isex_1 omitted)

i.inhalation1~s _Iinhalatio_1-9 (naturally coded; _Iinhalatio_1 omitted)

i.payorforreg~s _Ipayorforr_1-99 (naturally coded; _Ipayorforr_1 omitted)

i.LOSres _ILOSres_1-5 (naturally coded; _ILOSres_1 omitted)

i.yrgrp748085~c _Iyrgrp7480_1-7 (naturally coded; _Iyrgrp7480_1 omitted)

Median regression Number of obs = 6642

Raw sum of deviations 81149 (about 12)

Min sum of deviations 62245 Pseudo R2 = 0.2330

---------------------------------------------------------------------------

days1burnc~r | Coef. Std. Err. t P>|t| [95% Conf. Interval]

-------------+-------------------------------------------------------------

Age 6-15 | 1.636364 .5110511 3.20 0.001 .6345387 2.638189

Age 16-45 | 1.363636 .3649436 3.74 0.000 .6482291 2.079044

Age 46-65 | 4.181818 .460147 9.09 0.000 3.279782 5.083855

Age >65 | 6.636364 .7589585 8.74 0.000 5.14856 8.124167

TBSA% 21-40% | 21.09091 .4564286 46.21 0.000 20.19616 21.98566

TBSA% 41-60% | 42 .8492973 49.45 0.000 40.3351 43.6649

TBSA% >60% | 72.90909 1.439728 50.64 0.000 70.08676 75.73142

Non-White | .0909091 .3351282 0.27 0.786 -.5660503 .7478684

Female | .6363636 .2930815 2.17 0.030 .0618295 1.210898

Inhalation | 10.36364 .5947599 17.42 0.000 9.197715 11.52956

Medicare | 3 .6189611 4.85 0.000 1.786637 4.213363

Medicaid | .6363636 .3155409 2.02 0.044 .0178016 1.254926

Self-Pay | -.1818182 .4298459 -0.42 0.672 -1.024455 .6608185

WA not King | 2.727273 .2977475 9.16 0.000 2.143592 3.310954

Alaska | 8.727273 .8509715 10.26 0.000 7.059094 10.39545

Idaho | 6.636364 .947596 7.00 0.000 4.77877 8.493957

Montana | 6 .8912586 6.73 0.000 4.252846 7.747154

1980-1984 | -1.818182 .5855173 -3.11 0.002 -2.965984 -.670379

1985-1989 | -4.727273 .5724544 -8.26 0.000 -5.849468 -3.605077

1990-1994 | -5 .5739462 -8.71 0.000 -6.12512 -3.87488

1995-1999 | -5.727273 .5891097 -9.72 0.000 -6.882118 -4.572428

2000-2004 | -7 .5892269 -11.88 0.000 -8.155075 -5.844925

2005-2009 | -7.818182 .5795372 -13.49 0.000 -8.954262 -6.682102

_cons | 11 .5798143 18.97 0.000 9.863377 12.13662

---------------------------------------------------------------------------
