# Supplementary material for: Harborview Burns – 1974 to 2009
Source: PLoS One. 2012 Jul 5;7(7):e40086. doi: 10.1371/journal.pone.0040086 (PMC3390332; doi:10.1371/journal.pone.0040086)
Supplement: File S6 — Fluid Regression. This is the STATA regression of fluids/kg/% on opioid equivalents, benzodiazepines, tracheal intubation, central venous pressure lines, arterial lines, pulmonary artery catheters, and colloid. (DOC) [file pone.0040086.s006.doc]

Supporting File S6

STATA Regression 2 – First 24-Hour Fluids

xi: regress fluidskgpercent oe i.benzo i.intubated i.cvp i.artline i.pacath i.colloid, robust

i.benzo _Ibenzo_0-9 (naturally coded; _Ibenzo_0 omitted)

i.intubated _Iintubated_0-9 (naturally coded; _Iintubated_0 omitted)

i.cvp _Icvp_0-9 (naturally coded; _Icvp_0 omitted)

i.artline _Iartline_0-9 (naturally coded; _Iartline_0 omitted)

i.pacath _Ipacath_0-9 (naturally coded; _Ipacath_0 omitted)

i.colloid _Icolloid_0-9 (naturally coded; _Icolloid_0 omitted)

Linear regression Number of obs = 111

F( 7, 103) = 7.55

Prob > F = 0.0000

R-squared = 0.2666

Root MSE = 1.432

---------------------------------------------------------------------------

| Robust

First 24hr Fluids | Coef. Std. Err. t P>|t| [95% Conf. Interval]

-------------+-------------------------------------------------------------

Opioid Equivalents | .033267 .0139114 2.39 0.019 .005677 .060857

Benzodiazepines | -.3798799 .3430902 -1.11 0.271 -1.060318 .3005586

Intubation | .9999627 .4451489 2.25 0.027 .1171148 1.882811

CVP line | .1880922 .3823626 0.49 0.624 -.5702338 .9464183

Arterial line | .7008706 .4471308 1.57 0.120 -.185908 1.587649

PA Catheter | -.0792351 .4475914 -0.18 0.860 -.9669271 .808457

Colloid | .4839563 .3506319 1.38 0.171 -.2114393 1.179352

cons | 4.161193 .1886153 22.06 0.000 3.787119 4.53526

---------------------------------------------------------------------------
