# Supplementary material for: Harborview Burns – 1974 to 2009
Source: PLoS One. 2012 Jul 5;7(7):e40086. doi: 10.1371/journal.pone.0040086 (PMC3390332; doi:10.1371/journal.pone.0040086)
Supplement: File S7 — Mortality Regression Harborview. This is the STATA regression of mortality with standard admissions at Harborview on age, TBSA%, inhalation injury, gender, race/ethnicity and time period. (DOC) [file pone.0040086.s007.doc]

Supporting File S7

STATA Regression 3 – Mortality in Type 1 Admissions on Age, TBSA%, Inhalation, Gender, Race/ethnicity and Time Period.

xi: logistic LD i.agegrpmodMoreau i.tbsagrpmodGaleiras i.inhalation i.sex i.racegrp i.yrgrp748085etc if LD < 9 & agegrpmodMoreau < 9 & tbsagrpmodGaleiras < 9 & inhalation < 9 & sex < 9 & racegrp < 9 & type == 1, robust;

i.agegrpmodMo~u _Iagegrpmod_1-5 (naturally coded; _Iagegrpmod_1 omitted)

i.tbsagrpmodG~s _Itbsagrpmo_1-4 (naturally coded; _Itbsagrpmo_1 omitted)

i.inhalation _Iinhalatio_1-9 (naturally coded; _Iinhalatio_1 omitted)

i.sex _Isex_1-2 (naturally coded; _Isex_1 omitted)

i.racegrp _Iracegrp_1-9 (naturally coded; _Iracegrp_1 omitted)

i.yrgrp748085~c _Iyrgrp7480_1-7 (naturally coded; _Iyrgrp7480_1 omitted)

Logistic regression Number of obs = 9377

Wald chi2(16) = 1100.65

Prob > chi2 = 0.0000

Log pseudolikelihood = -1018.9254 Pseudo R2 = 0.5787

---------------------------------------------------------------------------

| Robust

LD | Odds Ratio Std. Err. z P>|z| [95% Conf. Interval]

-------------+-------------------------------------------------------------

Age 6-15 | .5047137 .1794765 -1.92 0.054 .2513954 1.013288

Age 16-45 | .7829661 .1843211 -1.04 0.299 .4935809 1.242017

Age 46-65 | 4.015486 .9592295 5.82 0.000 2.514206 6.413207

Age >65 | 28.09146 6.59454 14.21 0.000 17.73179 44.50369

TBSA% 21-40% | 11.11296 1.802419 14.85 0.000 8.086726 15.27168

TBSA% 41-60% | 41.38669 8.371026 18.41 0.000 27.84156 61.52164

TBSA% >60% | 444.2019 98.32461 27.54 0.000 287.8498 685.4802

Inhalation | 6.147453 .876887 12.73 0.000 4.648125 8.130413

Female | 1.675474 .2183873 3.96 0.000 1.297744 2.163148

Non-White | 1.289802 .2143734 1.53 0.126 .9312102 1.78648

1980-1984 | .8296119 .1891331 -0.82 0.413 .5306644 1.29697

1985-1989 | .671625 .1505207 -1.78 0.076 .432873 1.042061

1990-1994 | .621162 .1419404 -2.08 0.037 .3969161 .9721004

1995-1999 | .88263 .1839897 -0.60 0.549 .5865978 1.328058

2000-2004 | .7959046 .1753112 -1.04 0.300 .5168563 1.22561

2005-2009 | .3854073 .0831997 -4.42 0.000 .2524451 .5884002

---------------------------------------------------------------------------
