# Supplementary material for: Harborview Burns – 1974 to 2009
Source: PLoS One. 2012 Jul 5;7(7):e40086. doi: 10.1371/journal.pone.0040086 (PMC3390332; doi:10.1371/journal.pone.0040086)
Supplement: File S8 — Mortality Regression ABA-NBR. This is the STATA regression of mortality with standard admissions in the ABA-NBR on age, TBSA%, inhalation injury, gender, race/ethnicity and time period. (DOC) [file pone.0040086.s008.doc]

Supporting File S8

STATA Regression 4 – Regression ABA-NBR Mortality in Type 1 Admissions on Age, TBSA%, Inhalation, Gender, Race/ethnicity and Time Period

. xi: logistic dead_lhe019 i.agegrpmodMoreauv2 i.tbsagrpmodGaleirasv2 i.inhale019 i.gender019 i.race019 i.yrgrpregressv2 if mortregresset == 1 & type == 1, robust;

i.agegrpmodMo~2 _Iagegrpmod_1-5 (naturally coded; _Iagegrpmod_1 omitted)

i.tbsagrpmodG~2 _Itbsagrpmo_1-4 (naturally coded; _Itbsagrpmo_1 omitted)

i.inhale019 _Iinhale019_0-9 (naturally coded; _Iinhale019_0 omitted)

i.gender019 _Igender019_0-9 (naturally coded; _Igender019_0 omitted)

i.race019 _Irace019_0-9 (naturally coded; _Irace019_0 omitted)

i.yrgrpregres~2 _Iyrgrpregr_1-3 (naturally coded; _Iyrgrpregr_1 omitted)

Logistic regression Number of obs = 66116

Wald chi2(12) = 8133.74

Prob > chi2 = 0.0000

Log pseudolikelihood = -8852.428 Pseudo R2 = 0.4576

---------------------------------------------------------------------------

| Robust

dead_lhe019 | Odds Ratio Std. Err. z P>|z| [95% Conf. Interval]

-------------+-------------------------------------------------------------

Age 6-15 | .4731223 .0755926 -4.68 0.000 .3459184 .6471028

Age 16-45 | 1.855457 .1856271 6.18 0.000 1.525083 2.2574

Age 46-65 | 7.524197 .7639329 19.88 0.000 6.166483 9.180848

Age >65 | 46.41978 4.700318 37.90 0.000 38.06389 56.60999

TBSA% 21-40% | 6.365001 .3356417 35.10 0.000 5.740009 7.058044

TBSA% 41-60% | 25.05061 1.685857 47.86 0.000 21.95504 28.58265

TBSA% >60% | 142.1933 10.66603 66.09 0.000 122.7523 164.7132

Inhalation | 3.842698 .1758849 29.41 0.000 3.512981 4.203362

Female | 1.18875 .0527955 3.89 0.000 1.089648 1.296864

Non-White | 1.212993 .0548391 4.27 0.000 1.110134 1.325381

2000-2004 | 1.08343 .0566156 1.53 0.125 .9779588 1.200276

2005-2009 | .9811358 .0489915 -0.38 0.703 .8896633 1.082013

---------------------------------------------------------------------------
