# Supplementary material for: Harborview Burns – 1974 to 2009
Source: PLoS One. 2012 Jul 5;7(7):e40086. doi: 10.1371/journal.pone.0040086 (PMC3390332; doi:10.1371/journal.pone.0040086)
Supplement: File S9 — Mortality Regression Harborview rBaux. This is the STATA regress of mortality with standard admissions at Harborview on rBaux score, gender, race/ethnicity and time period. (DOC) [file pone.0040086.s009.doc]

Supporting File S9

STATA Regression 4 – Mortality in Type 1 Admissions on rBaux score, Gender, Race/ethnicity and Time Period.

. xi: logistic LD rBaux i.sex i.racegrp i.yrgrp748085etc > if LD < 9 & inhalation1no2yes < 9 & sex < 9 & racegrp < 9 & type == 1, robust;

i.sex _Isex_1-2 (naturally coded; _Isex_1 omitted)

i.racegrp _Iracegrp_1-9 (naturally coded; _Iracegrp_1 omitted)

i.yrgrp748085~c _Iyrgrp7480_1-7 (naturally coded; _Iyrgrp7480_1 omitted)

Logistic regression Number of obs = 9377

Wald chi2(9) = 885.27

Prob > chi2 = 0.0000

Log pseudolikelihood = -1004.4145 Pseudo R2 = 0.5847

---------------------------------------------------------------------------

| Robust

LD | Odds Ratio Std. Err. z P>|z| [95% Conf. Interval]

-------------+-------------------------------------------------------------

rBaux | 1.088666 .0031773 29.11 0.000 1.082456 1.094911

Female | 1.560187 .1911077 3.63 0.000 1.227192 1.983538

Non-White | 1.585693 .2538388 2.88 0.004 1.158665 2.170101

_Iyrgrp748~2 | .8386517 .183908 -0.80 0.422 .5456593 1.288966

_Iyrgrp748~3 | .6325437 .1399313 -2.07 0.038 .4100036 .9758732

_Iyrgrp748~4 | .5126119 .1112092 -3.08 0.002 .3350609 .7842482

_Iyrgrp748~5 | .7516588 .1526971 -1.41 0.160 .5047796 1.119282

_Iyrgrp748~6 | .6270884 .1297749 -2.25 0.024 .4179979 .94077

_Iyrgrp748~7 | .3251309 .0684435 -5.34 0.000 .2152144 .491185

---------------------------------------------------------------------------
